# Health benefits from risk information of air pollution in China

**DOI:** 10.1038/s41598-023-42502-6

**Published:** 2023-09-18

**Authors:** Zhaohua Wang, Jie Liu, Bo Wang, Bin Zhang, Nana Deng

**Affiliations:** 1 School of Management and Economics, Beijing Institute of Technology, Beijing, 100081 China; 2 Research Center for Sustainable Development and Intelligent Decision, Beijing Institute of Technology, Beijing, China

**Keywords:** Environmental sciences, Environmental social sciences

## Abstract

Risk-related information regarding air pollution can help people understand the risk involved and take preventive measures to reduce health loss. However, the health benefits through these protective behaviors and the health threat of information inequality have not been systematically measured. This article reports the health gains and losses caused by the interaction of “air pollution—air pollution information—human”, and studies the heterogeneity and impact of this interaction. Based on field investigations and transfer learning algorism, this study compiled the first nationwide city-level risk-related information (ERI) response parameter set in China. Then, we developed a Information-Behavioral Equivalent PM_2.5_ Exposure Model (I-BEPEM) model to project the health benefits caused by the impact of environmental risk-related information on residents’ protective behaviors under different scenarios. The protective behavior led by air pollution risk information reduces 5.7% PM_2.5_-related premature deaths per year. With a 1% increase in regional ERI reception, PM_2.5_-related premature mortality decreases by 0.1% on average; If the level of information perception and behavioral protection in all cities is the same as that in Beijing, PM_2.5_-related premature deaths will decrease by 6.9% annually in China. Further, changing the air quality standard issued by China to the American standard can reduce the overall PM_2.5_-related premature deaths by 9.9%. Meanwhile, compared with men, other age groups and rural residents, women, older persons, and urban residents are more likely to conceive risk information and adopt protective behaviors to reduce the risk of premature death from air pollution. Air pollution risk information can significantly reduce people's health loss. Changing the real-time air quality monitoring information indicator standard to a more stringent level can quickly and effectively enhance this effect. However, the uneven distribution of this information in regions and populations has resulted in the inequality of health gains and losses.

## Introduction

Exposure to air pollution leads to a wide variety of outcomes. Objectively judging the relative impact of these risks on personal and population health is fundamental to individual survival and societal prosperity. Many people suffer from premature deaths due to air pollution each year^1,2^, and its impact is particularly prevalent in developing countries^3^. Approximately, 1 million people die every year from air pollution (mainly due to PM_2.5_, PM_10_,_,_ and other particulate matter) in China^3–6^, causing economic losses of more than 100 billion US dollars^7,8^. Most of the existing literature discussed the attribution of premature death related to air pollution, such as climate change^9^, the usage of solid fuels in kitchen^10–12^, emissions from different industries^13–15^, expense, and income^16,17^. Further, the health impact of air pollution exposure on individuals over time and space have been identified^18–20^. Relevant policies and standards, such as the Clean Fuel Alternative Plan^21–24^, Comprehensive Clean Air Plan^25^, and expected air quality compliance on air-pollution-related deaths have been explored to solve this puzzle^26,27^.

Most existing studies have considered humans as passive receivers of pollution. The air pollution level that humans experience has been measured solely based on the level of ambient air pollution (AAP). However, humans do not passively endure the negative effects of air pollution; they take steps to limit or eliminate the health risk associated with the air pollution^28,29^. In this process, self-protection behavior modifies people’s activity pattern and subsequently reduces exposure to polluted air, either directly or indirectly. The accuracy of air pollution hazard identification will be inevitably affected by not considering people’s active protection behavior and will lead to overestimation of premature deaths. Additionally, different groups or regions have different accessibility to air-pollution-related information due to differences in economy, politics, culture and belief, social status, and so on, creating the so-called “digital divide” or information inequality^30–32^. The emergence of the internet and social networks serves to exacerbate this phenomenon. The accessibility to information about environmental risks such as air pollution differs across regions or populations. To address the potential social equity issues, the identification of potential health losses for disadvantaged populations^33–35^ due to information inequality is imperative.

After experiencing the widespread haze around 2013 in China^36,37^, people started taking initiatives to gather information about haze and to protect themselves from air pollution by wearing anti-smog masks, cancelling outings, and using air purification equipments^38–40^. Moreover, the government has issued a series of relevant measures to improve the status quo. One of the most significant changes is to reformulate air quality standards that includes harmful pollutants such as PM_2.5_ into the air quality evaluation system, and to mandate that each local government department releases the current air quality information to the public in real time through multiple channels^41^. Air quality monitoring and early warning information are readily available in everyday life (similar to weather information) based on which individuals can determine whether to take preventive steps based on the data released. However, the impact of air pollution information on protective behavior and health benefits has not been explored. Incorporating air pollution information and preventive behaviors into human health benefit evaluation will help in determining future strategies to reduce air-pollution-related premature mortality.

**Figure 1:**
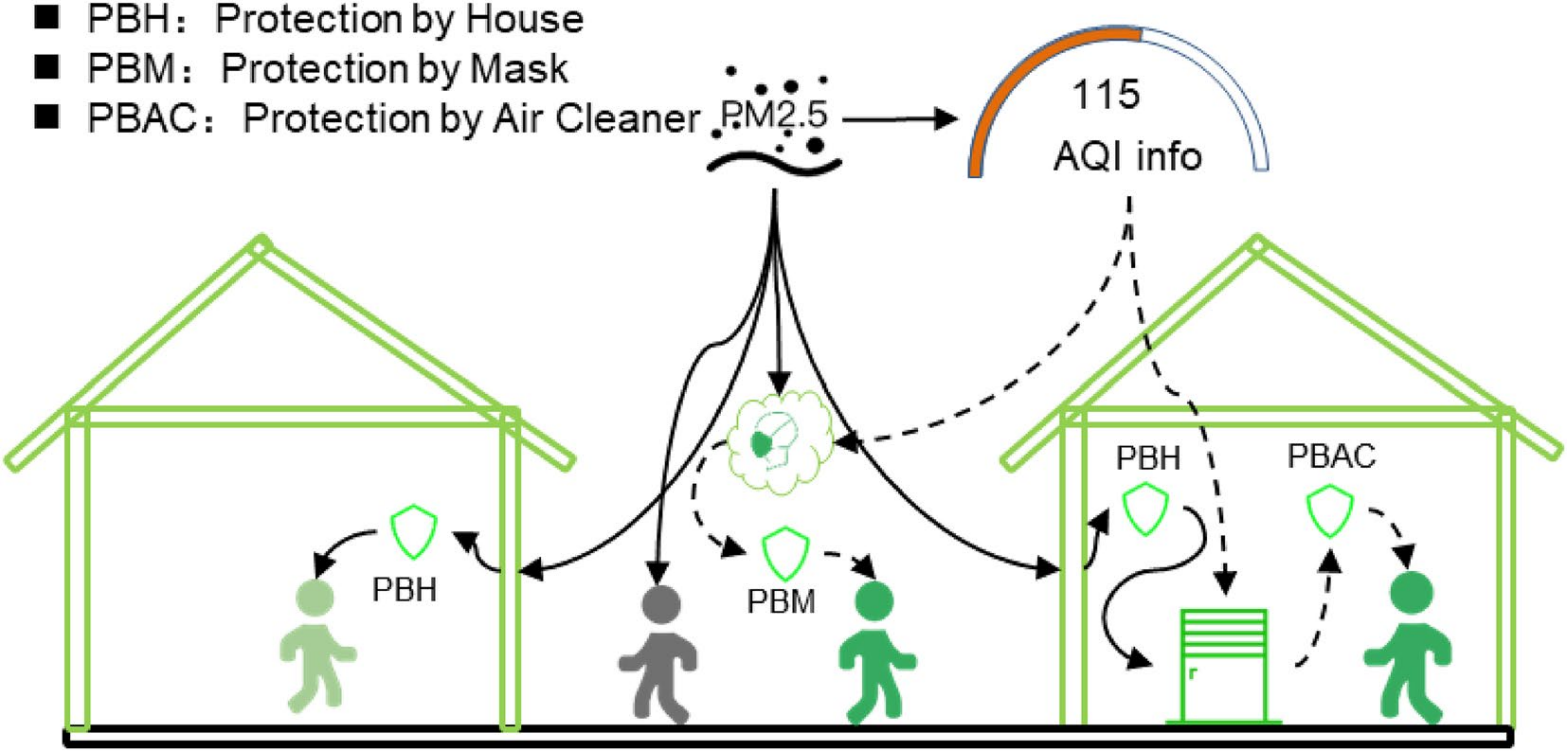
Effects of air pollution risk-related information on human exposure to PM_2.5._ AQI information represents the air pollution information, the green people represent receiving air pollution information and engaging in protective behavior, such as wearing a protective mask outdoors and activating air purification equipment (illustrated by a dashed line) when indoors, and the gray people represent the outdoor activities that are directly exposed to PM_2.5_. When indoors, all people, represented by light green and green, are shielded by buildings.

We developed the information-behavioral equivalent PM_2.5_ Exposure Model (I-BEPEM) to project the health benefits caused by the impact of environmental risk-related information (ERI) on residents’ protective behaviors under six different scenarios (Fig. 1 and Table 1). First, we analyzed the difference in PM_2.5_ exposure concentration caused by different behaviors of different populations under the influence of air pollution information (see Supplementary Material S1 for model settings). Second, to assess the relationship between perception of air pollution information and preventive behaviors, we compiled the first nationwide city level (294 cities) ERI behavior response parameter set in China based on field investigations and transfer learning algorism (Section “Urban protection data and inference”). Then, we used I-BEPEM (Section “Calculation of equivalent PM_2.5_”) to calculate equivalent PM_2.5_ exposure concentrations for different regions and groups. Finally, the integrated exposure–response (IER) model was adopted to quantify PM_2.5_-related premature deaths under each scenario. Furthermore, the health benefits brought by these protective behaviors and the health threat of information inequality have been discussed.

**Table 1:** PM_2.5_ exposure scenarios and related settings.

Scenarios	Settings
S0	Baseline scenario with ambient PM_2.5_ as exposure for all people (no protection)
S1	Based on scenario S0, the model adds indoor/outdoor activity time and protection by building (PBH)
S2	Based on scenario S1, the model includes people’s ideal protection from using mask and air cleaner (PBH, PBM, and PBAC)
S3	Based on scenario S2, the model includes people’s attention to AQI information release, leading to different degrees of protective behavior
S4	Based on scenario S3, the standards of air quality classification that used to publish air pollution monitoring information in China will be converted to US standards (adjusted AQI information)
S5	Based on scenario S3, the attention level and protection level of all cities are set to the same as Beijing (adjusted protections)

We found that the protective behavior led by conceived air pollution risk information decreased the number of PM_2.5_-related premature deaths by 5.7% per year (scenario S3 compared to scenario S1), which is approximately 41,000 lives in China. With a 1% increase in regional ERI reception, PM_2.5_-related premature mortality decreases by 0.1% on average, which is economically significant. When all cities will achieve the same degree of information perception and behavioral protection as Beijing (scenario S5), the average yearly PM_2.5_-related premature death will decline by 6.9%. Transforming China’s air quality forecast’s standard to the American standard (scenario S4) can reduce PM_2.5_-related premature mortality by 9.9%. Moreover, disparities in protective behavior among populations have resulted in a disparity in health benefits. Compared with men, other age groups and rural residents, women, older persons, and urban residents are more likely to conceive risk information and adopt protective behaviors to reduce the risk of premature death due to air pollution. Reportedly, this is the first study to incorporate ERI and protective behavior into health loss estimates, which provides a consistent way to understand and evaluate the disproportionally distributed ERI’s impact on regional and group health, which is fundamental for a tailored policy toward a sustainable future.

## Data and methods

### Urban protection data and inference

We designed a questionnaire to obtain the cognitive and protective behaviors data of different regions and groups about air pollution. After a strict quality control (including deleting some samples with obvious logical errors, missing data, and inconsistent addresses), we finally received 1072 valid questionnaires (see Supplementary Figs. 2–6 for the initial statistical information of some important indicators in the questionnaire). This study was approved by the Ethics Committee of the Beijing Institute of Technology (No. 22-1-103). All procedures performed in this study were in accordance with the 1964 Helsinki declaration and its later amendments or comparable ethical standards. All participants are allowed to fill in the questionnaire only when they understand the purpose of the survey and agree to the publication of the research results. And, online informed consent was obtained from all participants.

The settings of the core variables are as follows:

- ATTR_i:_ The attention ratio (ATTRi) is the proportion of people in different groups i (such as region, gender, and age) who pay attention to air pollution information. This data represents the statistical values of all samples in the survey questionnaire. For each respondent, we will inquire about the frequency of their daily attention to air pollution. There are 5 options for this question, with frequencies ranging from lowest to highest being most no, occasionally, generally, often, and most every day. When respondents with a frequency of often or above are marked as 1, otherwise it is 0. The group marked as 1 is considered to be concerned about air pollution information. In this way, by aggregating different groups, we can calculate the proportion of people in different groups who pay attention to air pollution information.
- MR_i_, CODR_i_, and ACR_i_: The three variables are whether they will wear masks or cancel going out in the air polluted weather (not pandemic period), and whether they have air purification equipment in the workplace and residential areas. If the answer to each question is “Yes,” select 1, otherwise 0. These variables are also used according to the ratio formed after group aggregation: the rate of group i wearing masks (MR_i_), canceling going out (CODR_i_), and having indoor air purification equipment (ACR_i_, the average of the rate of air purification equipment in workplaces and residential areas).
- ODR_i_: The proportion of outdoor activity time is mainly to investigate the average daily outdoor activity hours of individuals during the non-epidemic period, and then to calculate the outdoor activity proportion (ODR_i_) of group i.

To extrapolate the questionnaire results to all prefecture-level cities, we introduced transfer learning method into our work (see Supplementary material S3). The idea of transfer learning is to use the similarity of data, task type, or models to apply the models and knowledge learned in the old fields to the new fields. Including the problems and data in this paper, the final required prediction results are calculated as follows:

*Step 1* Align provincial statistical characteristic data (source domain) with urban characteristic data (target domain) by CORAL algorithm^42^:

1 $$\begin{array}{c}{D}_{\mathrm{s}}={\left[{F}_{s1}^{T},{F}_{s2}^{T}\dots {F}_{sm}^{T}\right]}_{m\times n}\end{array}$$

2 $$\begin{array}{c}{D}_{\mathrm{t}}={\left[{F}_{t1}^{T},{F}_{t2}^{T}\dots {F}_{tm}^{T}\right]}_{m\times k}\end{array}$$

3 $$\begin{array}{c}{{C}_{s}=\Sigma }_{s}+eye\left(m\right)\end{array}$$

4 $$\begin{array}{c}{{C}_{t}=\Sigma }_{t}+eye\left(m\right)\end{array}$$

5 $$\begin{array}{c}{D}_{\mathrm{s}}^{new}={D}_{s}*{C}_{s}^{-\frac{1}{2}}*{C}_{t}^\frac{1}{2}\end{array}$$

Equations (1) and (2) represent the feature datasets of the source domain and target domain, respectively; ${F}_{m}^{T}$ is the m^th^ feature of the dataset, where the source domain feature data are provincial statistical data from China Statistical Yearbook 2020^43^, and the target domain feature data are urban statistical data from China Urban Statistical Yearbook 2020^44^. The source domain and target domain have the same type of statistical indicators, including 18 indicators in the fields such as economics, environment, education, and population structure. As these indicators differ greatly at the city level and provincial level, we divide all indicators by the total population of the current region to obtain the per capita value of each indicator so that the characteristic scales of the source domain and target domain are the same.

*Step 2* Use the transformed source domain data to establish a supervised machine learning model and train it and use the trained model to predict the city-level variables.

**Figure 2:**
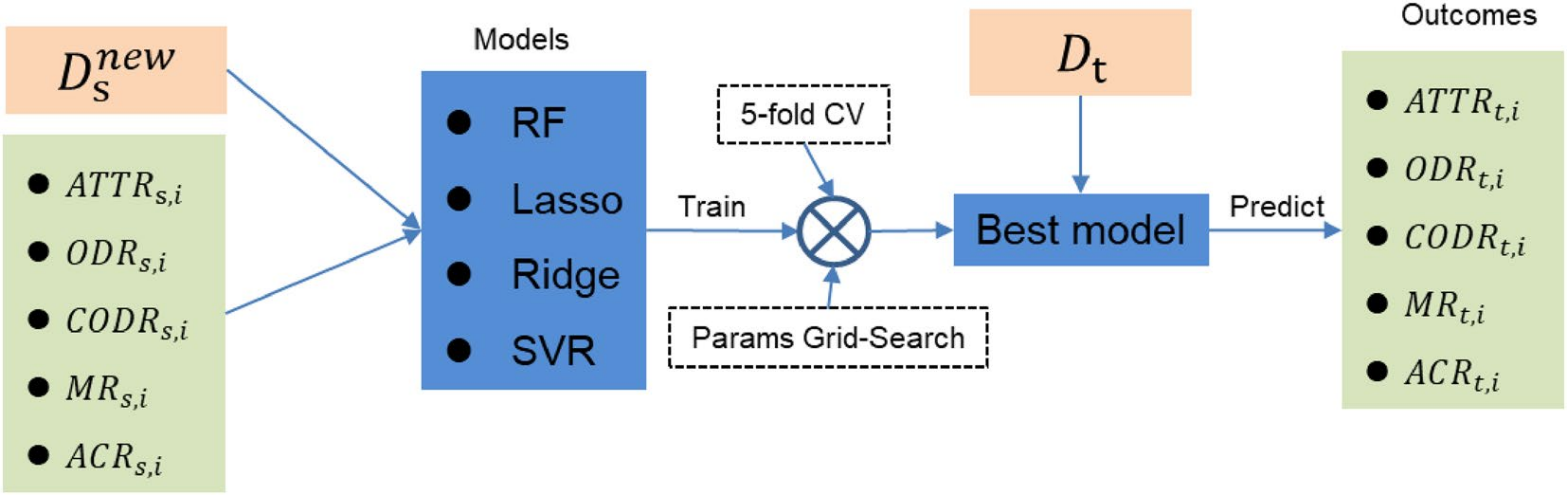
Model training and prediction process. RF, Random Forest; SVR, support vector machine.

The model architecture is shown in Fig. 2. ${D}_{\mathrm{s}}^{new}$ is the feature of input data that includes the five variables, which are the five tasks’ goal of training model, respectively. We selected four machine learning models as our candidate models: random forest, Lasso regression, Ridge regression, and support vector machine. These models are simple and efficient in structure, and their easy-to-use regularization technology limits the occurrence of overfitting. In the training process, the grid search method is used to automatically select the best super parameter for each task’s model. The fivefold cross-validation method is used to verify the accuracy of each model. Then, we select the model with the best performance in each task, and finally predict the corresponding variables of each city with city-level dataset (${D}_{\mathrm{t}}$).

According to the cross-validation and test results of the model, the validity and accuracy of our model are established (see Supplementary Material S5 and Table 1). Considering age, gender, and urban and rural groups, we used the total original questionnaire to calculate the variables of each group (see of Supplementary Material S5 and Table 2).

### Calculation of equivalent PM_2.5_

This research refers to the integrated population weighted exposure (IPWE) model created by Shen et al.^45^ and enhances it accordingly. The IPWE model distinguishes between household air pollution (HAP) and outside ambient air pollution (AAP) and incorporates people’s activity patterns into the model. We added outdoor PM_2.5_ permeability and people’s protective behavior led by risk information to the model (see Supplementary Material S4) and developed the I-BEPEM to assess people’s real PM_2.5_ exposure concentration.

Equation 6 expresses the I-BEPEM model based on the previous assumptions. The urban attention ratio and the protective behavior ratio are obtained from the prediction results of Section “Urban protection data and inference”, and both follow the $N({\mu }_{i}, {\theta }^{2})$ distribution. ${\mu }_{i}$ is the indicator’s forecast data for city i, and $\theta$ is the indicator’s standard deviation. ${\mathrm{pm}}_{i,t}$ represents the average concentration of PM_2.5_ in city i on day t. This indicator is derived from the data of over 2000 monitoring sites for surface air quality in China’s Ministry of Ecology and Environment^46^. The air quality index for city i on day t is denoted by $AQ{I}_{i,t}$. $IEP{E}_{i}$ is the annual equivalent comprehensive PM_2.5_ exposure value for city i. $threshold$ is the AQI value at which the air quality level of “lightly polluted” is reached. $DM$ is the mask’s protective effect or the PM_2.5_ attenuation rate after being filtered by the mask. The protective effect conforms to the Chinese government-issued group standard F9053 for “PM_2.5_ protective masks”^47^. According to Xiang et al.^48^, $D{H}_{i}$ represents the protective impact of buildings in various areas or the attenuation rate of PM_2.5_ in the outer environment when it penetrates a room. $DAC$ is the purification efficiency of air purification equipment, or the rate of PM_2.5_ concentration attenuation after air purification equipment has cleansed indoor air. This information is derived from the existing relevant measured data^49–52^. We consider the mean of these studies as the decay rate value. To ensure uncertainty, we assume that all types of decay rate data have a normal distribution, with the mean serving as their survey or reference value (see Supplementary Material S2 and for the corresponding variance settings).

6 $$\left\{\begin{array}{l}\begin{array}{l}OD{R}_{i}=OD{R}_{i}*(1-COD{R}_{i}*ATT{R}_{i})\\ M{R}_{i}=M{R}_{i}*ATT{R}_{i}\\ AC{R}_{i}=AC{R}_{i}*ATT{R}_{i}\\ IEP{E}_{AAP,i}=\frac{1}{T}\left\{\begin{array}{l}{\sum }_{t=1}^{T}{\mathrm{pm}}_{i,t}*OD{R}_{i}*\left(M{R}_{i}*DM+1-M{R}_{i}\right), if\,AQ{I}_{i,t}>Threshold\\ {\sum }_{t=1}^{T}{\mathrm{pm}}_{i,t}*OD{R}_{i},\,else\end{array}\right.\\ IEP{E}_{HAP,i}=\frac{1}{T}\left\{\begin{array}{l}{\sum }_{t=1}^{T}{\mathrm{pm}}_{i,t}*\left(1-OD{R}_{i}\right)*D{H}_{i}*\left(AC{R}_{i}*DAC+1-AC{R}_{i}\right), if\,AQ{I}_{i,t}>Threshold\\ {\sum }_{t=1}^{T}{\mathrm{pm}}_{i,t}*\left(1-OD{R}_{i}\right)*D{H}_{i}, else \end{array}\right.\\ IEP{E}_{i}= \, IEP{E}_{AAP,i}+IEP{E}_{HAP,i}\text{.}\end{array}\end{array}\right.$$

Table 2 displays the settings for several indicators for scenarios S0–S5. “Yes” indicates that the actual value of the indicator should be maintained. The values 0 and 1 denote the setting index value. “No” indicates that the indicator is not considered. According to our survey results, residents generally refer to the overall air quality level, rather than being limited to the AQI value of PM_2.5_. Residents are only likely to take protective measures when the air pollution level reaches “light polluted” (AQI > 100) or above. Both China and the United States regard the highest AQI value of all pollutants at each moment as the current overall AQI value and designate it as the primary pollutant^41,53^. According to the overall AQI value, the current air quality is divided into six levels: excellent, good, lightly polluted, moderately polluted, heavily polluted, and severely polluted. The difference is that when the PM_2.5_ concentration is less than 150 μg/m^3^ and PM_2.5_ is the primary pollutant, China’s AQI value may be lower than that of the United States (see Supplementary Material S10). Therefore, we map the Chinese air quality level to the new air quality level and AQI value based on the PM_2.5_ level in the US standard. In summary, we will use 100 as the threshold for AQI in our model. The protection level parameter for Beijing residents is set to Column S5 with the subscript BJ.

**Table 2:** Indicator settings in different scenarios.

Index	S0	S1	S2	S3	S4	S5
$ATT{R}_{i}$	0	0	1	Yes	Yes	$ATT{R}_{BJ}$
$OD{R}_{i}$	1	Yes	Yes	Yes	Yes	$OD{R}_{BJ}$
$COD{R}_{i}$	0	0	Yes	Yes	Yes	$COD{R}_{BJ}$
$M{R}_{i}$	0	0	Yes	Yes	Yes	$M{R}_{BJ}$
$AC{R}_{i}$	0	0	Yes	Yes	Yes	$AC{R}_{BJ}$
$DM$	0	0	Yes	Yes	Yes	Yes
$D{H}_{i}$	0	Yes	Yes	Yes	Yes	Yes
$DAC$	0	0	Yes	Yes	Yes	Yes
Threshold	No	No	100	100	35	100

### Premature death estimation

This study mainly uses the IER model developed by Burnett et al. and GBD 2019 disease data to estimate PM_2.5_-related premature death. IER model is widely recognized and uses PM_2.5_ concentration-related premature death risk estimation model^54^, and its calculation method is shown in Eq. 7.

7 $$\begin{array}{*{20}l} {RR_{IER} \left( z \right) = \left\{ {\begin{array}{*{20}l} {1 + \alpha \left( {1 - e^{{ - \gamma \left( {z - z_{cf} } \right)^{\delta } }} } \right),} & {if z > z_{cf} } \\ {1,} & { else} \\ \end{array} .} \right.} \\ \end{array}$$

Among them, z represents the annual mean equivalent PM_2.5_ concentration calculated for each city in Section “Calculation of equivalent PM_2.5_”. ${z}_{cf}$ is the minimum PM_2.5_ concentration with additional risk.$\alpha$, $\gamma$, and $\delta$ are computed by fitting this equation. This paper focuses primarily on the four major causes of premature PM_2.5_ mortality, namely ischemic heart disease (IHD), stroke, chronic obstructive pulmonary disease (COPD), and lung cancer (LC). The ${z}_{cf}$, $\alpha$, γ, and $\delta$ parameter values corresponding to the above four diseases are from Institute for Health Metrics and Evaluation (IHME). Each disease contains 1000 sets of parameter simulations. The final calculation method of PM_2.5_-related premature death for each city is shown in Eq. 8:

8 $$\begin{array}{c}{AC}_{i,k}=\frac{{RR}_{i,k}-1}{{RR}_{i,k}}\times {B}_{k}\times {P}_{i},\end{array}$$

where ${AC}_{i,k}$ and ${RR}_{i,k}$ are the number of PM_2.5_-related additional deaths and the relative risk of disease k in the ith city or group, respectively. ${B}_{k}$ is the basal incidence of disease k, which is from GBD 2019^4^. ${P}_{i}$ is the total population of the city or group i. To obtain interval estimates of PM_2.5_-related premature death, 1000 Monte Carlo simulations were performed for all parameters.

### Reduction amount of premature death and distribution of environmental risk information

Weibo (China’s equivalent to Twitter) and Baidu Index are the two main sources of ERI. Sina Weibo is the largest open social networking platform in China. It was founded in 2009 and had 450 million monthly active users and 250 million daily active users by 2018^55^. Baidu is the largest search engine in China. Through distributed crawler technology, the public application program interfaces (APIs) of these two platforms were searched for content containing environment-related keywords, as shown in Supplementary Table 3. After information extraction, cleaning, and conversion, approximately 2.3 million original microblogs related to the environment were obtained. These microblogs were forwarded approximately 140 million times and more than 30 million people participated in the discussion during 2013–2020. In addition to the Weibo data, we received the daily-level search index data for 294 cities during 2013–2020 as a supplement. We used all environment-related Weibo reposts and originals from different regions and Baidu search index as the total distribution of regional environmental information. Equation (9) defines the per capita access to ERI:

9 $$\begin{array}{c}ER{I}_{i}=\frac{1}{{P}_{i}}\sum_{T+1}^{T+t}\left({W}_{i,t}+{B}_{i,t}\right),\end{array}$$

where ${W}_{i,t}$ and ${B}_{i,t}$ are the total number of original and reposted environment-related microblogs and the search index in city i at time t, respectively. The time range is $[T+1, T+t]$. ${P}_{i}$ is the total population of city i.

The relationship between the reduction of premature death and the distribution of ERI is shown in Eq. (10).

10 $$\begin{array}{c}DDP10{k}_{i}=\beta \cdot ER{I}_{i}+{\sum }_{k}{\gamma }_{k}{X}_{k,i}.\end{array}$$

$DDP10{k}_{i}$ is the PM_2.5_-related premature deaths reduced by active protection per 10,000 people in city i. ${X}_{k,i}$ denotes the kth covariate of the ith city. All variables are log transformed. ${\gamma }_{k}$ is the coefficient of the kth covariate; β is our target coefficient, representing the percentage change in $DDP10{k}_{i}$ for every 1% change in ERI.

## Results

### Comprehensive equivalent PM_2.5_ exposure concentration

**Figure 3:**
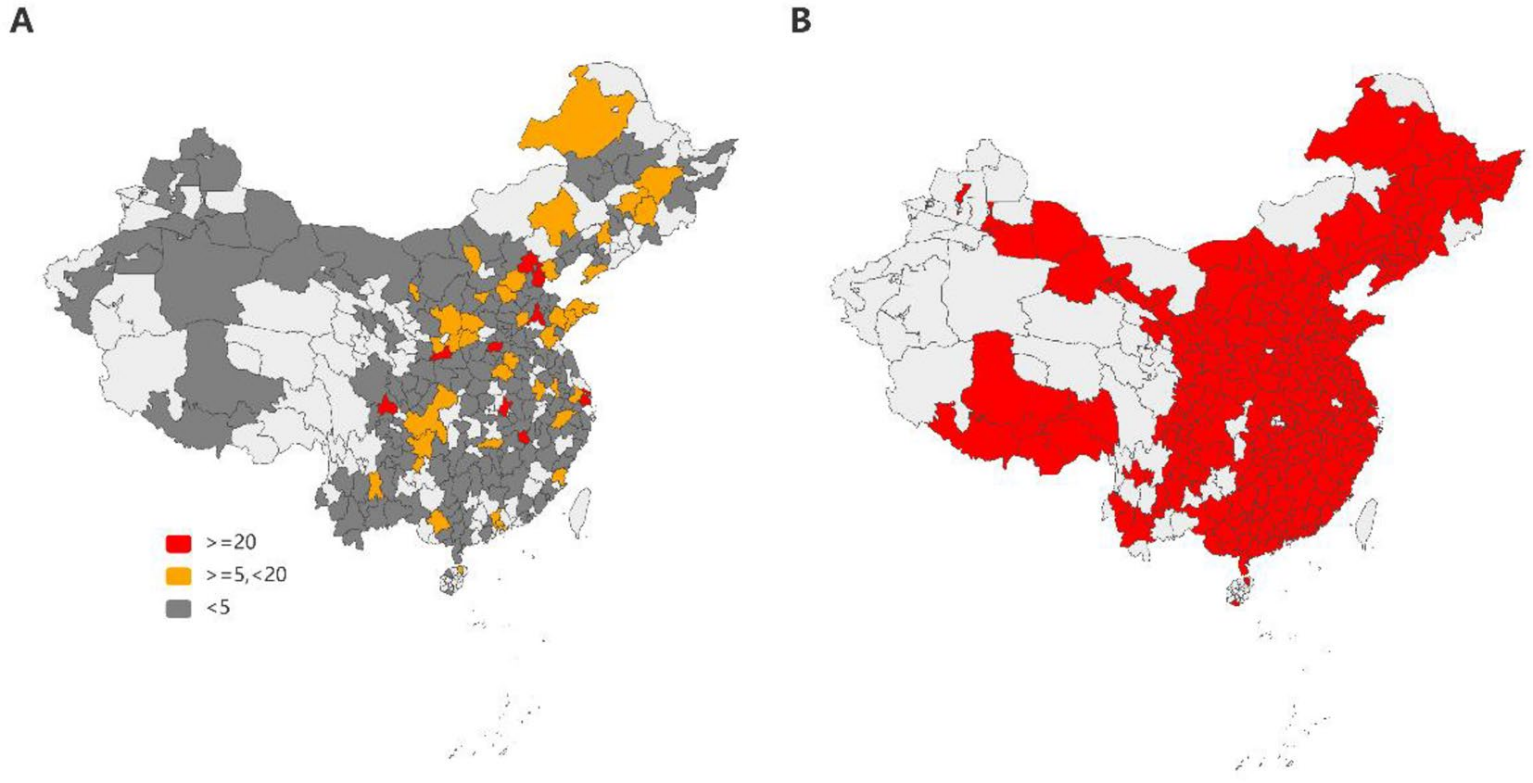
The spatial distribution of data. A is the questionnaire survey data. B is city statistics data. Those areas that are not covered by color have no data for the time being. The maps were drawn by Python (v3.10, https://www.python.org/) and Pyecharts (v2.0.3, https://pyecharts.org/#/), based on the Vector Border Map of China’s City level Administrative Division in 2021 (Geographic Coordinate System: CGCS_2000).

We compiled the nationwide ERI behavior response parameter set for 294 cities in China based on questionnaires and transfer learning algorism (Section “Urban protection data and inference”). The spatial distribution of questionnaire data is shown in Fig. 3A. The data samples of the questionnaire survey involved 236 cities, including 186 cities with a sample size less than 5 and only 9 cities with a sample size greater than 20. There are 294 cities’ city statistics (including economic, educational, medical and air quality statistics) data, and their spatial distribution is shown in Fig. 3B. Provincial statistics data include all 31 provinces except Hongkong, Macau and Taiwan Province. With these indicators, we simulated the comprehensive equivalent PM_2.5_ exposure concentration under scenarios S0–S5 by I-BEPEM model (Section “Calculation of equivalent PM_2.5_”).

**Figure 4:**
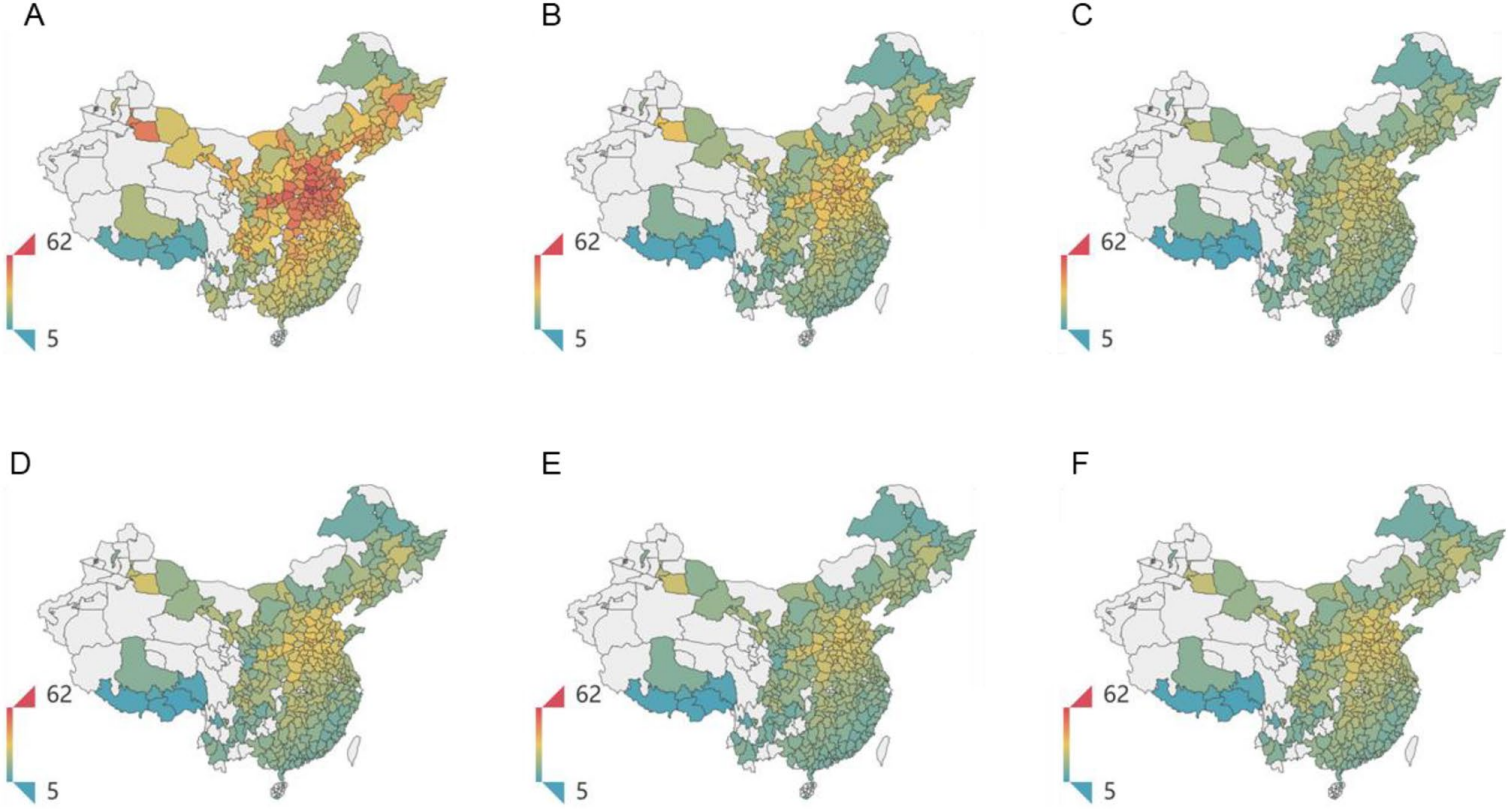
Distribution of PM_2.5_ exposure concentration in 294 prefecture-level cities in 2020 under scenarios S0–S5. The color from green to red represents the concentration value from low to high. The blank part of the map lacks relevant statistical data, and the proportion of this part of the population in the total is less than 10%; therefore, it is not considered. The maps were drawn by Python (v3.10, https://www.python.org/) and Pyecharts (v2.0.3, https://pyecharts.org/#/), based on the Vector Border Map of China’s City level Administrative Division in 2021 (Geographic Coordinate System: CGCS_2000).

The distribution of PM_2.5_ exposure concentration in 294 prefecture-level cities in 2020 is shown in Fig. 4. Figure 4A shows the spatial distribution of basic scenario S0. We observe that China’s air pollution events mainly occur in northern cities, where highly polluting and energy-intensive enterprises are clustered; Fig. 4B–F shows that the equivalent PM_2.5_ concentration under scenarios S1–S5 has decreased to varying degrees compared with scenario S0. This indicates that the differences in population activity patterns and protective behaviors in different regions have a direct impact on their PM_2.5_ exposure concentrations.

**Figure 5:**
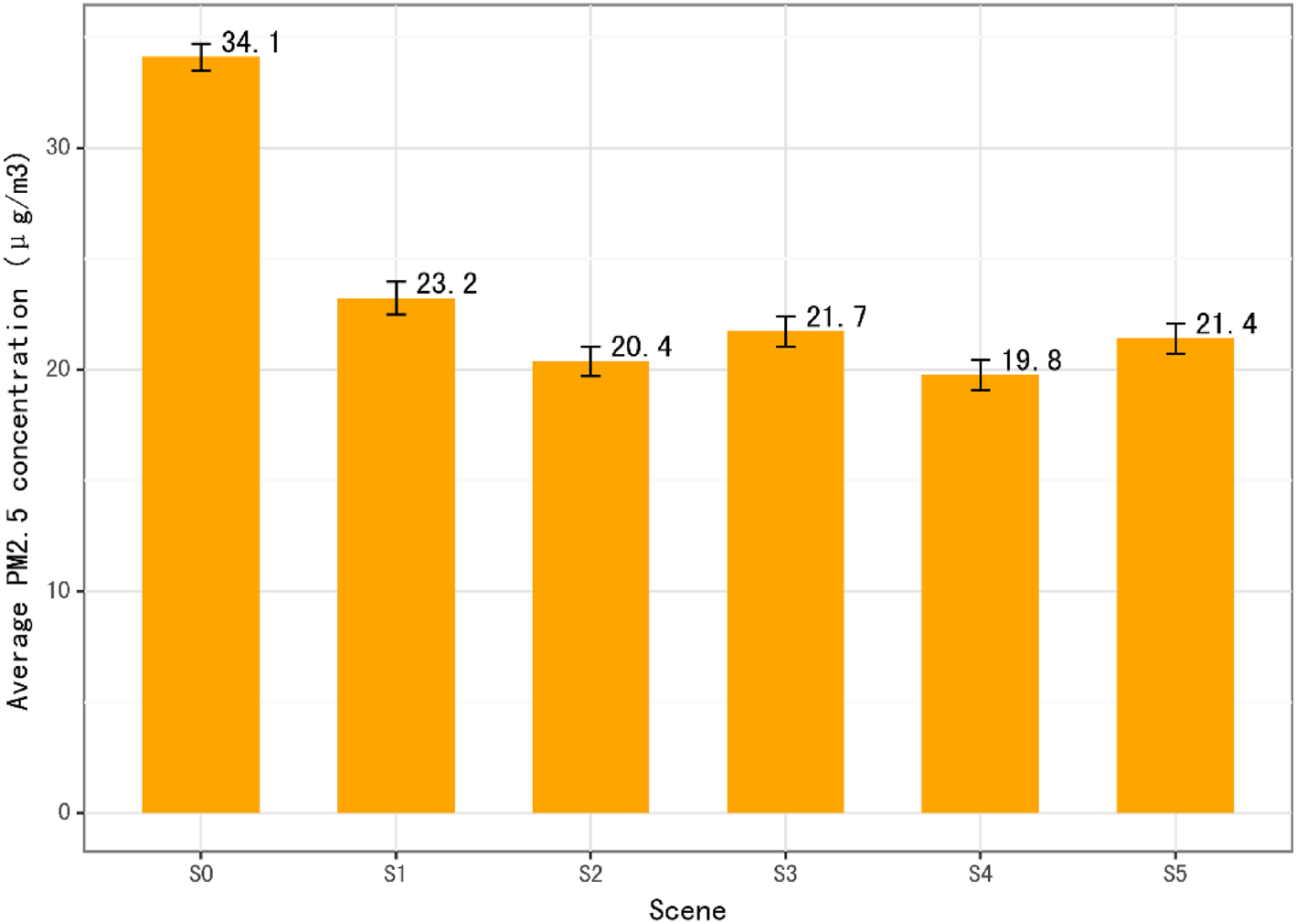
Average equivalent PM_2.5_ exposure concentration of 284 prefecture-level cities under scenarios S0–S5 (μg/m3).

The average equivalent PM_2.5_ exposure concentration values under different scenarios are shown in Fig. 5. The average ambient PM_2.5_ concentration under scenario S0 is 34.1 μg/m^3^, and the concentration under scenarios S1–S5 decreases by 10.9 μg/m^3^, 13.7 μg/m^3^, 12.4 μg/m^3^, 14.3 μg/m^3^, and 12.7 μg/m^3^, respectively, compared with scenario S0. Scenario S4 has the largest decline, which indicates that the different levels of air pollution information release can significantly affect people’s PM_2.5_ exposure concentration. Compared with the ideal protection behavior (S2), S3 is more realistic with an exposure concentration of 1.5 μg/m^3^, which is higher than that of S2, since people can only take protective actions when they are aware of the degrees of air pollution. When the level of risk information reception and perception in each city is the same as Beijing (S5), the average exposure concentration will decrease by 0.3 μg/m^3^ compared with S3, which indicates that the level of risk information release and perception in China on average are significantly lower than that of Beijing, which provide evidence regarding the underlying risk caused by information inequality.

### Overall health benefits under different scenarios

According to the simulated results of PM_2.5_ exposure concentration, we used IER model (Sections “Calculation of equivalent PM_2.5_” and “Premature death estimation”) to calculate the number of premature deaths of different scenarios. The total number of premature deaths of each scenario is composed of premature deaths caused by four PM_2.5_-related diseases: stroke, IHD, COPD, and LC.

**Figure 6:**
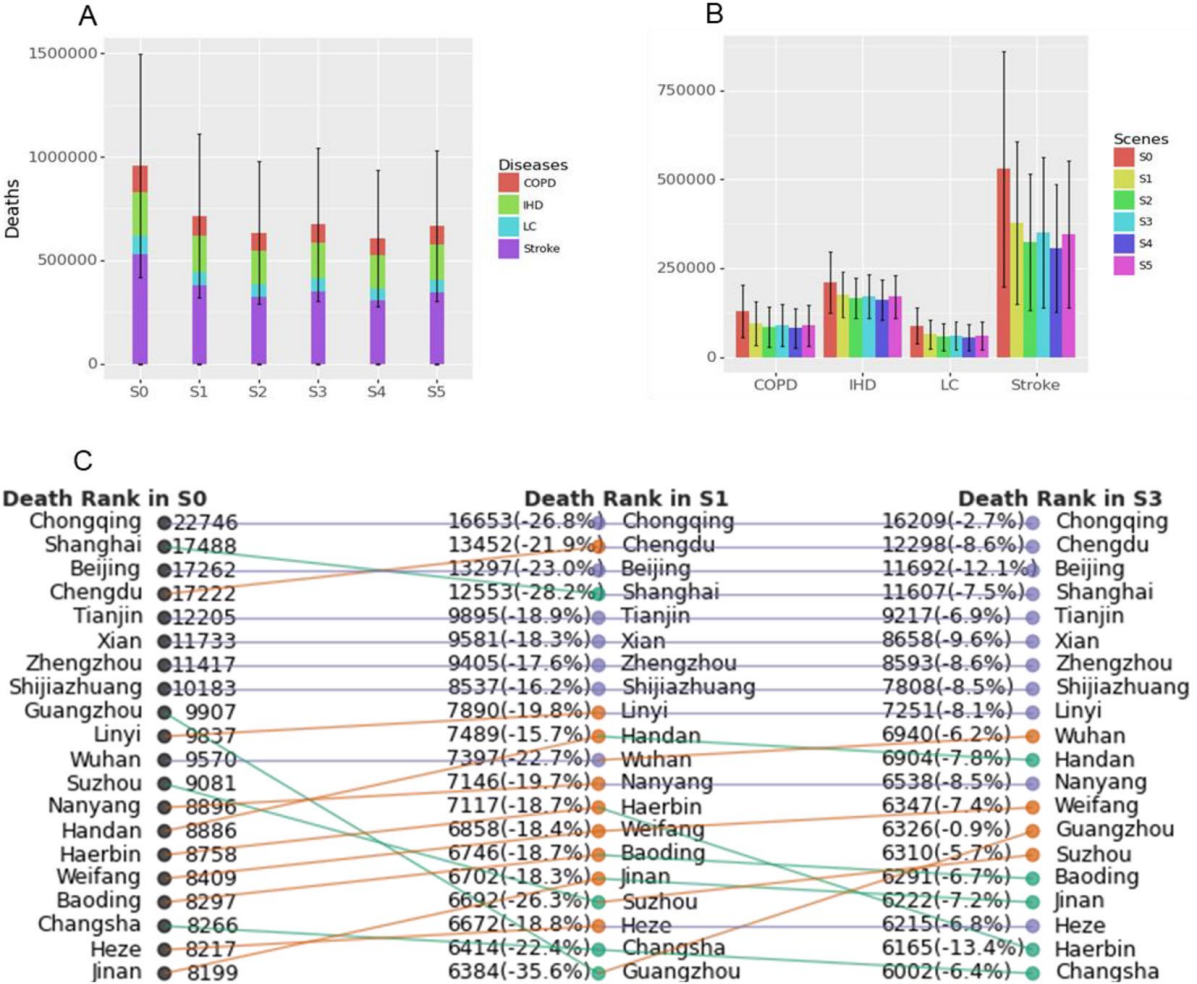
Estimate of PM_2.5_-related premature deaths. This includes excess death caused by lung cancer (LC), chronic obstructive pulmonary disease (COPD), ischemic heart disease (IHD), and stroke, with a confidence level of 95%. A is the number of premature deaths and the proportion of four diseases under different scenarios. B is the number of premature deaths of four diseases in different scenarios. C is the ranking of the top 20 cities with premature deaths in the S0 scenario when they transition from scenario S0 to S1 and from S1 to S3, as well as the magnitude of the change rate in premature deaths.

Figure 6A shows that under the baseline scenario S0, the premature death related to environmental PM_2.5_ in China is about 0.958 (0.417–1.500: 95% confidence interval, the same below) million in 2020; when considering the pattern of human activity (S1), the PM_2.5_-related premature death is about 0.715 (0.318–1.112) million, with an average decrease of about 25.4% compared with the baseline scenario. Under scenario S2, adding people’s ideal protective behavior to the model, about 84,000 premature deaths can be reduced, which is 11.7% lower than scenario S1. Considering that people’s air pollution information attention adjusted actual prevention behavior, the number of related premature deaths is 6.8% higher than that of scenario S2 [0.674 (0.303–1.043) million]. The gap between S3 and S2 is the result of the difference between people’s protective behavior and practical action. Nevertheless, scenario S3 is still about 5.7% lower than that of scenario S1 with a reduction of 41,000 premature deaths. Considering the regional differences in air pollution information release and its induced protective behavior difference, if we set the level of attention and protection of other regions to the same as Beijing, it can reduce 6.9% of premature deaths annually (compare to S1), and a further decrease of 9,000 people compared with scenario S3. When the air quality standard of China’s air quality forecast information is changed to that of the America’s (scenario S4), the number of premature deaths is about 0.607 (0.278–0.935) million, which is about 9.9% lower than that of scenario S3, and an additional 67,000 premature deaths can be reduced, which is about 15.1% lower than S1, and about 108,000 premature deaths are expected to be exempted.

There are positive effects of air pollution information and events on people’s cognitive and protective behaviors that have reduced many premature deaths, and an inequality in this effect (scenario S5) has been evidenced. Furthermore, there is a significant gap between the recognition and perception of air pollution information and the adoption of actual protective actions (scenarios S2 and S3). A more stringent air quality standards can fill this gap (scenario S4).

Figure 6B shows the number of premature deaths caused by various PM_2.5_-related diseases under different scenarios. It is observed that the number of premature deaths under different scenarios maintains the same order, which is stroke, IHD, COPD, and LC from high to low. The average proportion of the four disease is 52.4%, 25.0%, 13.5%, and 9.1%, respectively.

Figure 6C shows the ranking changes of the top 20 cities in scenario S0 with the highest premature deaths. Under the benchmark scenario (S0), regions with large populations and relatively serious pollution level generally rank high, such as Chongqing, Shanghai, Beijing, Chengdu, and Tianjin. However, under scenarios S1 and S3, the ranking of premature deaths in these regions has changed significantly. The difference in people’s activity patterns in different regions reflected in S1 significantly affected the PM_2.5_ exposure concentration per capita with a 15.7–35.6% decline in premature deaths. Considering the influence of information (S3), the top 10 cities with the highest death basically remain unchanged; however, the average decline of the death (8.0%) under S3 is greater than that of the bottom 10 cities (6.9%). This indicates that people’s protection awareness and behavior in areas with more serious air pollution are higher than those in areas with less air pollution, thus avoiding more premature deaths. Even in cities with similar severity of air pollution, such as Beijing and Chongqing, the number of avoided premature deaths are different, that is, it has decreased by 9.4% more in Beijing than that in Chongqing. This indicates that people’s awareness and protective behavior are affected by not only the level of actual risk but also the differences in risk information perception and cognition caused by the imbalance of economic and cultural development. The spatial distribution of premature death can be seen in Supplementary Fig. 7.

In order to investigate the robustness of our study under different premature death computational models, we used the GEMM model^56^ as an example to recalculate some of the results in this study. The results indicate that, overall distribution pattern of premature deaths in different scenarios is completely the same for the two models. This means that using other models will not invalidate any conclusions in this study. See detail information in Supplementary Materials S9.

### Information inequality and premature death related to PM_2.5_

To evaluate the impact of information inequality on PM_2.5_-related premature death, we used Baidu Search Index and Sina Weibo data (same as Google Search Index and Twitter in China, Section “Reduction amount of premature death and distribution of environmental risk information”) characterized by the inequality degree of receiving ERI in 294 cities in China through Gini coefficient. Then, we analyzed the magnitude of the marginal impact of information inequality on regional premature deaths through regression (Section “Reduction amount of premature death and distribution of environmental risk information”).

**Figure 7:**
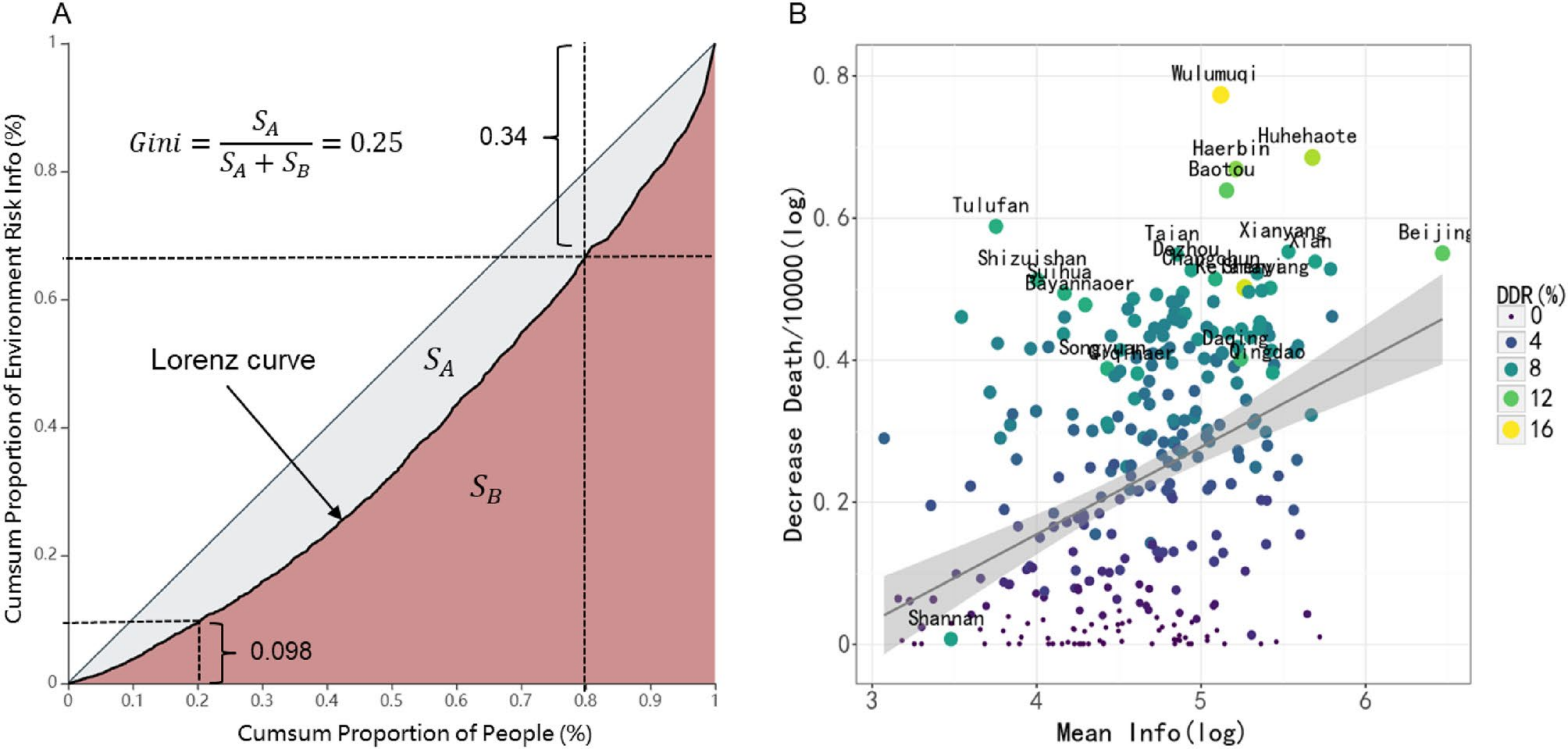
Inequality curve and relationship between ERI and DDP10K. A is the Lorentz curve of the distribution of environmental-related information dissemination in the city. The vertical axis is the proportion of the accumulated information obtained in the total number, and the horizontal axis is the proportion of the corresponding population in the total number, which is arranged from low to high. B is the relationship between the amount of information received per capita (ERI) related to the environment of different cities and the amount of PM_2.5_-related premature death reduction per 10,000 people (DDP10k) in cities. The size of the point represents the amount of reduction rate of premature death (DDR) caused by protective behaviors. Both the vertical and horizontal axes are logarithmic results.

Figure 7A shows the inequality degree of ERI spread among 294 cities’ residents from 2019 to 2020. The top 20% of residents with the largest amount of ERI release occupy 34% of the total, while the lowest 20% only occupy 9.8% of the total. The Gini coefficient is 0.25, which indicates obvious information inequality. Figure 7B shows the relationship between the number of premature deaths avoided per 10,000 (DDP10k, the scenario S3 compare with S1) and the amount of ERI per capita in each city. We observe that there is an obvious positive relationship between them.

We use econometric analysis to quantify the marginal impact of ERI on DDP10k. The model (1) in Table 3 is the benchmark model with ERI as the independent variable; model (2) is the regression result after adding a series of control variables, which is our target model (see Section “Reduction amount of premature death and distribution of environmental risk information”). The result of model (2) shows that information inequality could significantly affect PM_2.5_-related premature death; every 1% increase in city per capita ERI access can significantly reduce 0.105% (*p* < 0.01) PM_2.5_-related premature deaths per 10,000 people.

**Table 3:** The relationship between DDP10k and ERI.

	Dependent variable:	
	DDP10k	
	(1)	(2)
ERI	0.109***	0.105***
	(0.014)	(0.014)
Second industry ratio		0.001
		(0.001)
Hospital beds		0.232***
		(0.046)
Population		− 0.081*
		(0.048)
GDP		0.003
		(0.041)
Green ground		0.017
		(0.031)
Government general spend		− 0.043
		(0.032)
Industry company		− 0.052***
		(0.017)
Income		− 0.100*
		(0.057)
PM_2.5_		0.008***
		(0.001)
Constant	− 0.270***	1.226**
	(0.065)	(0.566)
Observations	294	294
R2	0.171	0.561
Adjusted R2	0.168	0.546
Residual Standard Error	0.164 (df = 292)	0.121 (df = 283)
F Statistic	60.269*** (df = 1; 292)	36.166*** (df = 10; 283)

*p < 0.1, **p < 0.05, and ***p < 0.01.

### Heterogeneity of health benefits among different groups

**Figure 8:**
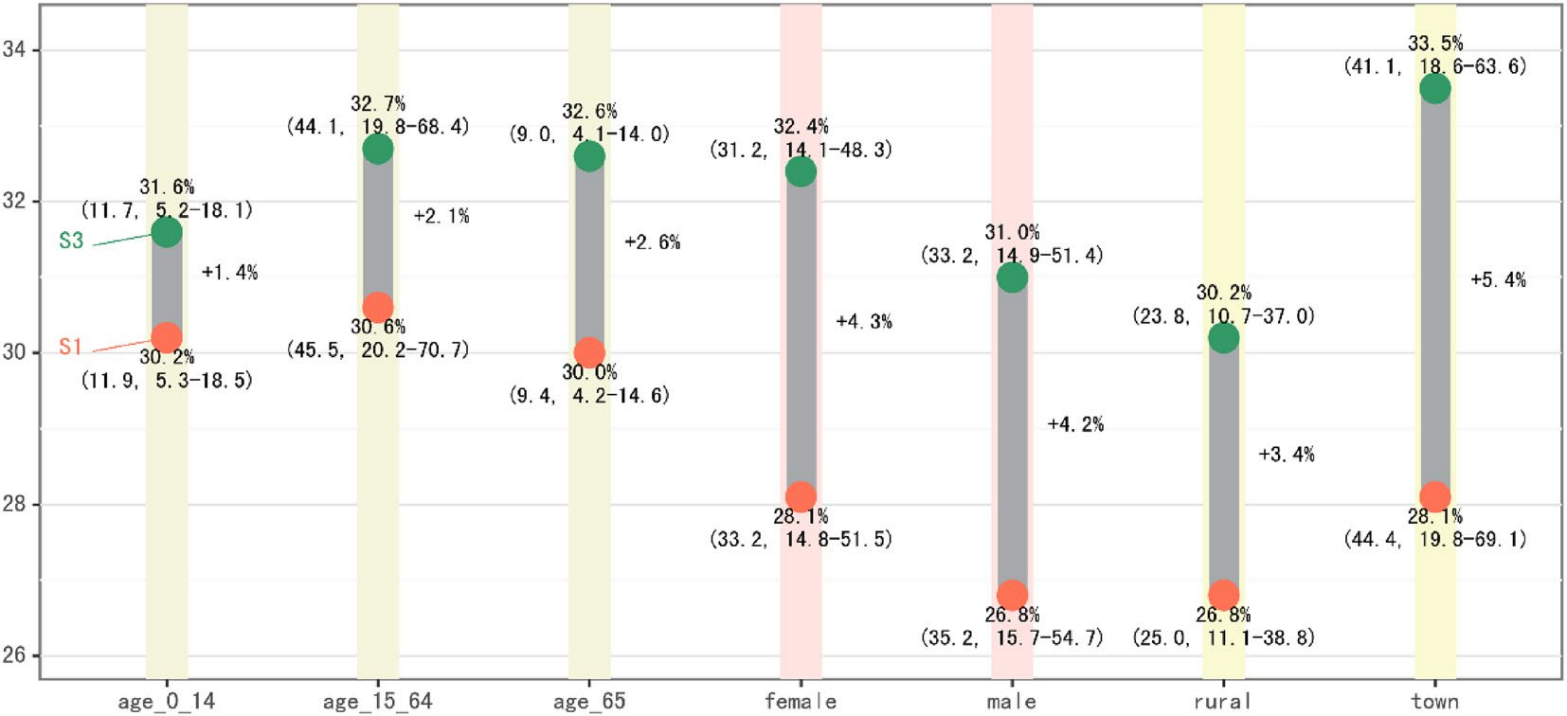
Changes in the number of premature deaths of scenarios S3 and S1 compared with S0 in different group. The green scatter is the change percentage of S3, the red scatter is the change percentage of S1, and the figures in brackets are the number of premature deaths related to PM_2.5_ (10,000) and its 95% confidence interval. The length of the gray column is the percentage point of the change of scenario S3 relative to S1.

There are disparities in the number of premature deaths among different demographic groups since their activity patterns and self-protective behaviors are different. As shown in Fig. 8, the number of premature deaths in the juvenile group (0–14 years old), the young and middle-aged group (15–64 years old), and the older persons group (over 65 years old) under scenario S3 was 117,000 (52–181, 95% CI), 441,000 (198–684, 95% CI), and 90,000 (41–140, 95% CI), respectively. Under the scenario S1, the daily activity patterns of the young and middle-aged group avoided more premature deaths (30.6%); the older persons’ group perform more outdoor activity; however, they paid more attention to protective behaviors, and thus PM_2.5_-related premature deaths decreased by about 2.6% in S3. In terms of gender, the number of premature deaths in scenario S3 is 312,000 (141–483, 95% CI) for females and 332,000 (149–514, 95% CI) for males. Females are also more willing to take protective actions than males, which makes the number of premature deaths of women decrease by 0.1% more than that of men. In terms of urban and rural areas, there are considerable disparities. Although the number of premature deaths related to PM_2.5_ in rural areas under scenario S3 is only 57.9% than that in urban areas due to urban and rural population distribution, compared with scenario S1, the number of premature deaths decrease degree rate among rural residents is 2.0 points lower than that of urban residents under scenario S3. The number of premature deaths avoided by the activity patterns in urban areas under scenario S1 is 1.3 points higher than that in rural areas, which indicates that rural residents are at a disadvantage both in terms of direct exposure to PM_2.5_ and self-protection level.

## Discussion and conclusion

This study incorporates both human protective awareness and behavior in PM_2.5_-related premature mortality estimation. I-BEPEM model has been established to quantify the influence of risk information on protective behavior in lowering PM_2.5_-related premature death, which is a new benchmark for rediscovering and evaluating the disproportionally distributed pollution-related health benefits. This study provides a detailed projection of how people’s behavior changes in response to air pollution, illustrates the health benefits of people’s “self-protective” behavior under the influence of ERI, and examines the inequity involved. The results showed that when the protective behavior led by air pollution risk information was incorporated, the number of premature deaths decreased by approximately 5.7%, which approximately avoided 41,000 people’s premature death. Moreover, disparities in health gains and losses between urban and rural areas have been exacerbated by disparities in health awareness and behavior. In terms of premature death reduction rates led by risk information, the older person’s group (2.6%) is higher than the adolescent group (1.4%) and the young and middle-aged group (2.6%); the female group (4.3%) is slightly higher than the male group (4.2%), and the urban population (5.4%) is significantly higher than the rural population (3.6%). Further research establishes that information inequality is a significant driver of the disparity in health benefits and losses associated with PM_2.5_ among regions. For every 1% increase in regional ERI release, there is a 0.1% decrease in PM_2.5_-related premature deaths per 10,000 persons on average.

Our results have important implications, not only for China but also for any country or region that seeks protection for its citizen from pollution, in a climate with potentially increasing hazards. While developing the economy and protecting the environment, we should also pay special attention to the premature death induced by information inequality. This inequality exists not only among regions but is also prevalent among subgroups of demographic. Ignoring this inequality will result in increased health losses and exacerbate the inequality in social development. Governments should prioritize enhancing the scientific education and publicity in disadvantaged areas. Furthermore, the hidden health losses led by the relative low pollution threshold standard have a negative impact on how the local population perceives the risk information of air pollution from air pollution monitoring agency and take protective actions. Raising local air pollution regulations for monitoring and early warning could be the quickest and least expensive way to avoid health loss, compared to energy-saving remodeling plans and renewable energy plans that involve significant personnel and material resources. Finally, a 6.8% gap between people’s willingness to protect themselves from air pollution and their real protective activities have been evidenced. Effectively increasing public risk awareness could be a crucial strategy for bridging this gap.

This study has the following limitations. First, the protective behavior data used in this study are primarily derived from online questionnaires. Even if the penetration rate of the internet users in China surpasses 90% (WeChat has around 1.29 billion monthly active users)^57^, it may lead to potential choice bias issues. Future studies should combine online and offline methods. Second, this study disregards the influence of indoor pollution sources, which prior research has found to be concentrated in the northern rural areas^24,45^. Therefore, this study’s estimates of premature deaths in rural regions may be underestimated. Lastly, although our study is conducted during the COVID-19 outbreak, it does not consider lockdowns and mandatory mask orders. This may have led to an overestimation of the premature mortality toll caused by PM_2.5_. It should be highlighted that despite the aforementioned flaws in this study, the validity of the conclusions remains unaffected. Future research should concentrate on addressing the aforementioned flaws to enhance the estimation accuracy of the number of early deaths attributable to PM_2.5_.

### Supplementary Information

Supplementary Information.

## Data Availability

We upload the data on GitHub from https://github.com/MR3118/Health-benefits, requiring the password at *20221108*. All data, figures and models are processed in Python, R and office suit. Any additional information required to reanalyze the data reported in this paper is available from Bo Wang.
