# Supplementary material for: Health benefits from risk information of air pollution in China
Source: Sci Rep. 2023 Sep 18;13:15432. doi: 10.1038/s41598-023-42502-6 (PMC10507042; doi:10.1038/s41598-023-42502-6)
Supplement: Supplementary file 1 — Supplementary Information. [file 41598_2023_42502_MOESM1_ESM.docx]

Health benefits from risk information of air pollution in China

# S1 Basis for formulation of analysis framework

According to the risk perception theory, people's risk prevention behavior is a dynamic process, and the level of personal awareness of air pollution risk is mainly affected by family, social and cultural factors and previous experience. When faced with the relevant risk event again, the assumption of the risk event will be activated; Then, people will have the intention and behavior of protection^1^. When individuals or groups have protective behaviors against air pollution, it is equivalent to reducing their exposure concentration of PM2.5 and other particles in the air pollution environment. The factors affecting PM2.5 exposure mainly include the distribution concentration of environmental PM2.5 in time/space (indoor/outdoor), personal attention to air pollution risk information, willingness to take protective measures, etc. We quantified the above factors through air quality data and more than 1000 questionnaires. According to the questionnaire survey, about 84.8% of the respondents believed that the air pollution weather such as smog had a great impact on their health, which showed that the public had a full understanding of air pollution. About 37.1% of the residents believed that the air pollution in their living areas was relatively serious, and about 42.2% would often pay attention to the air pollution. This shows that although most people are aware of the harm of air pollution, people just do not pay too much attention to it. From Supplementary Figure 1, we can find that there is a significant positive relationship between people's cognition, perception and concern about air pollution and their protective behaviors.


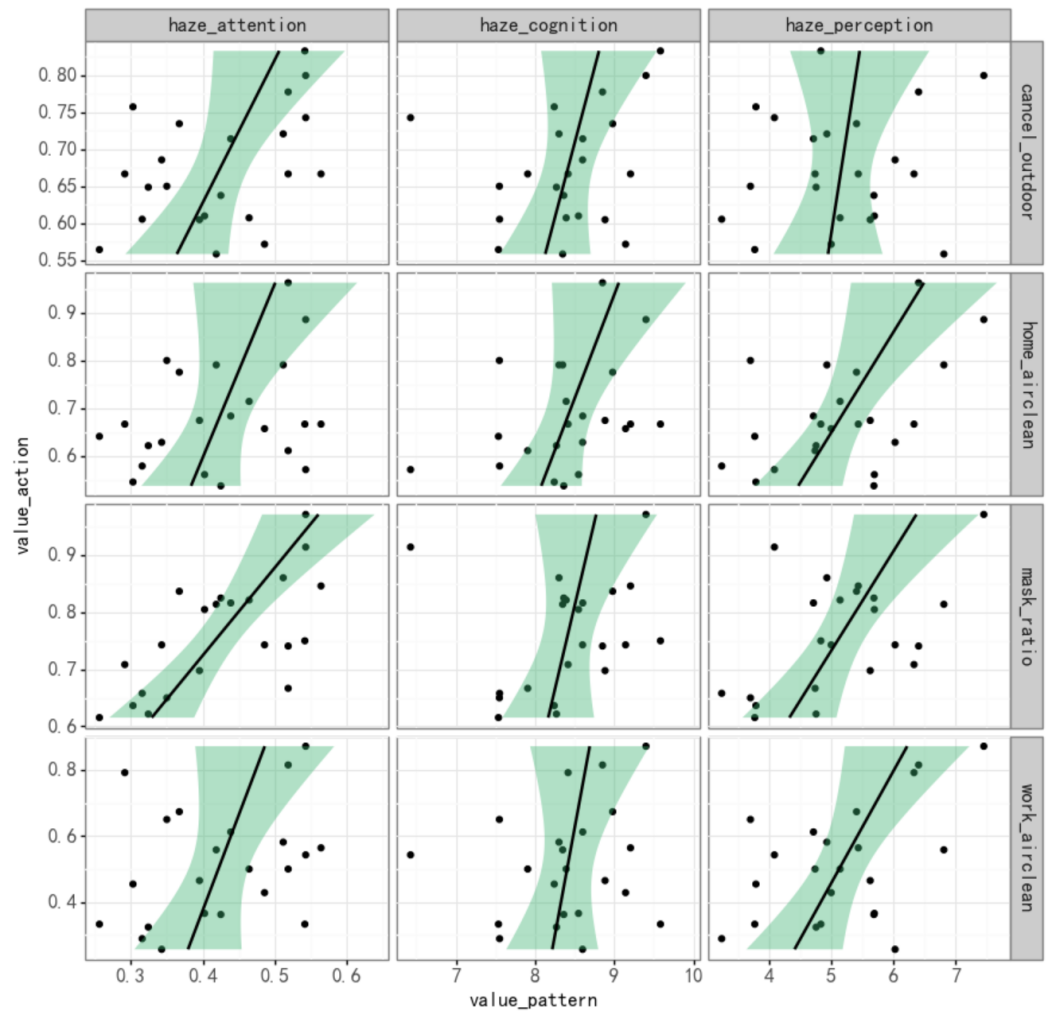


**Supplementary Figure 1.** The relationship between people's awareness, perception and concern about air pollution and their protective behaviors. The points are aggregated by province. The cognitive level and perception level are 0-10, and the attention is the proportion of people who pay attention to air pollution problems frequently and almost every day. Among the protective behaviors, the cancellation of going out, wearing a mask and using an air purifier (home/workplace) are all proportional values.

Based on the above results and analysis, we have established a comprehensive equivalent PM2.5 exposure model (IEPEM) as shown in Figure 1, which shows the difference of PM2.5 exposure concentration of different populations in air pollution weather. Our questionnaire shows that about 82.6% of the respondents obtain the current air pollution degree through air quality forecast information on the Internet, television and other media. They can be divided into high-sensitive groups and low-sensitive groups according to their sensitivity to air pollution problems such as haze. Among them, the high-sensitive groups pay more attention to the air pollution weather information and take corresponding protective measures, such as canceling go out or reducing the time outdoor, wearing masks, purchasing and using air purifier equipment, etc.; Low-sensitive groups are hardly affected by the information of air pollution. For the high-sensitive population, the exposure concentration is equivalent to the environmental PM2.5 concentration which is attenuated by the impact of protection factors (POH, POM, POAC), while the low-sensitive population is directly exposed to environmental PM2.5 when they are outdoors, and only protected by buildings when they are indoors.

# S2 Analysis of sources of uncertainty

In the estimation process of premature death related to PM2.5 in this paper, the uncertainties mainly come from the following four aspects: First, the data of behavior indicators related to protection. For example, the rate of wearing masks, the rate of canceling going out, the installation rate of air purification equipment, the rate of going out time, the degree of concern, etc. The first-hand data of these indicators are nationwide survey data, but because the questionnaire is difficult to cover the whole country, we introduced a machine learning model to extrapolate the above indicators to solve this problem. Due to the inherent error of the estimation model, although the prediction accuracy is high, it is still inevitable to introduce uncertainty. In order to measure the impact of this error on the uncertainty of estimation results, we regard the estimated values of each indicator as variables with the following normal distribution.

$$\begin{aligned} Index_{i,k}=N\left( \mu_{i,k},\sigma_{k}^{2} \right)\#\left( 1 \right) \end{aligned}$$

$Index_{i,k}$ is the k-th variable related to urban protection, and $\mu_{i,k}$ is the predicted value of the k-th protection related index of city i, $\sigma_{k}$ is the mean absolute error (MAE) of the k-th index prediction value. Second, the protective effect of various protective measures on air pollution. We have referred to the group standard F90 of "PM2.5 mask" issued by the Chinese government for the protective effect of masks, that is, the filtering effect of PM2.5 is greater than 90%^2^; The protection effect of buildings in different regions, namely the attenuation rate of outdoor environment PM2.5 when it penetrates into the room, is derived from the study of Xiang et al. ^3^; The purification efficiency of air purification equipment comes from the relevant measured data^4-7^. The average values of these measured values are taken as the unified attenuation rate values in this paper. Because the above results inevitably have errors in practice, we use the same method to introduce the above indicators into the calculation model as normal distribution variables with variance of 0.1. Third, the daily concentration of PM2.5 in each city. The PM2.5 concentration used in this paper is the hourly level data of ground monitoring stations in each city. Considering that there are differences in PM2.5 concentration values in different regions and at different times in the same city, we assume that the PM2.5 concentration value of city i is a normal distribution subject to the mean value and variance of the 24-hour PM2.5 concentration of the same day. Fourth, IER model error. The risk estimation model of premature death is IER model, which is developed by Burnett et al. ^8^. We also used the 1000 groups of parameters provided by them as simulation parameters of the model. Using the above settings, we conducted 1000 Monte Carlo simulations on the number of premature deaths in each city.

# S3 Extrapolation of questionnaire results

As we all know, the data obtained from the questionnaire is generally limited, especially in a country with a population of more than 1 billion, such as China. Previous research on air pollution related protective behaviours was generally limited to a certain city, or a limited number of samples were used to represent the entire population of each province. Although this method is reasonable in some case, it is obviously rough and can’t reflect the high regional heterogeneity of different cities. Because the weather and air quality forecast information are generally aggregated at the city level, and there are certain differences in the cognition and perception of air pollution info among urban residents, the protection behaviours in this paper are set to be city level inevitability. But the questionnaire data obviously cannot cover all cities or its sample size does not have statistical significance. In our questionnaire, the number of cities with statistical significance in the sample is even less than that in the province. A feasible plan is to establish a machine learning prediction model with the provincial questionnaire results as the prediction items, and other provincial statistical data as the auxiliary features, and then use the city level statistical data with the same meaning as the model input to predict the city level attention and protection behaviour data. However, this method still has potential defects: the joint probability distribution of city level data and provincial level data may be different, which does not conform to the principle of data independent and identically distributed (i.i.d) in machine learning. In order to extrapolate our questionnaire results to all prefecture level cities, we introduced transfer learning method into our work. The idea of transfer learning is to use the similarity of data, task type or models to apply the models and knowledge learned in the old fields to the new fields. The unified representation can be as follows^9^:

$$\begin{aligned} f^{*}=argmin\frac{1}{N_{S}}\sum_{i=1}^{N_{s}} l\left( v_{i}f\left( x_{i} \right), y_{i} \right)+\lambda R\left( T\left( D_{s} \right),T\left( D_{t} \right) \right)\#\left( 2 \right) \end{aligned}$$

$N_{S}$ is the number of samples in the source domain, $v_{i}$ is the sample weight of the source domain, $l\left( \cdot, \cdot\right)$ is the empirical loss function, $R\left( \cdot, \cdot\right)$is the transfer regularization term, and $T\left( \cdot, \cdot\right)$ is the source domain $D_{s}$ and target domain $D_{t}$ Characteristic transformation function. According to different parts of optimization formula (1), transfer learning can be divided into sample weight transfer method, feature transformation transfer method, pre-training model transfer method, etc. The transfer learning method used in this paper is the subspace feature transformation method^10^. This method usually assumes that the source domain and target domain data will have similar distribution in the transformed subspace. The data distribution is aligned by aligning the statistical characteristics of the data, which is applicable to the unsupervised migration of the target domain without labels, and is consistent with the extrapolation task in this paper. CORAL (Correlation Alignment) algorithm^11^ is a relatively mature method in the subspace transformation migration method, which is also the specific method applied in this paper. CORAL algorithm mainly aligns the second-order features of two domains to achieve the alignment of feature data distribution. Assume that $C_{s}$ and $C_{t}$ are the covariance matrix of the source domain and the target domain respectively. If A is the matrix to be solved, the optimization goal is:

$$\begin{aligned} \min_{A}\left| \left| A^{T}C_{s}A-C_{t} \right| \right|_{F}^{2}\#\left( 3 \right) \end{aligned}$$

# S4 Base of I-BEPEM

This research refers to the Integrated Population Weighted Exposure (IPWE) model created by Shen et al.^12^ and enhances it accordingly. The IPWE model distinguishes between household air pollution (HAP) and outside ambient air pollution (AAP) and incorporate people's activity patterns into the model. As stated in Equation (4), the proportion of people's activity time in different areas is used as the total weight of PM_2.5_ concentration in each space.

$$\begin{aligned} E=C_{0}+\sum_{m=1}^{m} t_{m}\left( C_{m}-C_{0} \right) \#\left( 4 \right) \end{aligned}$$

$C_{0}$ refers to the concentration of PM_2.5_ in the outdoor atmosphere, $t_{m}$ is the residence time in space m, and $C_{m}$ is the concentration of PM_2.5_ in space m. The IPWE approach is more reasonable and precise than many earlier studies that directly used the ambient PM_2.5_ concentration as the PM_2.5_ exposure concentration of inhabitants, hence it has been extensively adopted. Nonetheless, the model contains the following flaws: The model equates indoor PM_2.5_ concentration with the total of outdoor ambient PM_2.5_ and indoor pollution sources. However, numerous studies have demonstrated that the outdoor PM_2.5_ cannot enter the room unimpeded and will attenuate^13,14^. Consequently, this paper incorporates the PM_2.5_ permeability coefficient computed by Xiang et al.^3^ into the enhanced model. People's protective behaviours, such as wearing masks, canceling going out, purchasing and using air purification equipment in haze weather, will have a direct impact on people's PM_2.5_ exposure concentration. The above information enables us to establish the foundation of the I-BEPEM model. Before building this model, the following assumptions are made:

-  Hypothesis 1: The activity patterns, protective behaviours, and attention behaviours of each city and group are independent and conform to a normal distribution.
-  Hypothesis 2: Individuals will determine whether to take precautionary activities only after getting information about air pollution.
-  Hypothesis 3: Only when air pollution reaches a specific threshold will people adopt precautionary measures. In this paper, the air pollution level is selected as " lightly polluted", that is, the air quality index (AQI) is greater than the minimum AQI value at the corresponding level.

# S5 Distribution of main data in the questionnaire


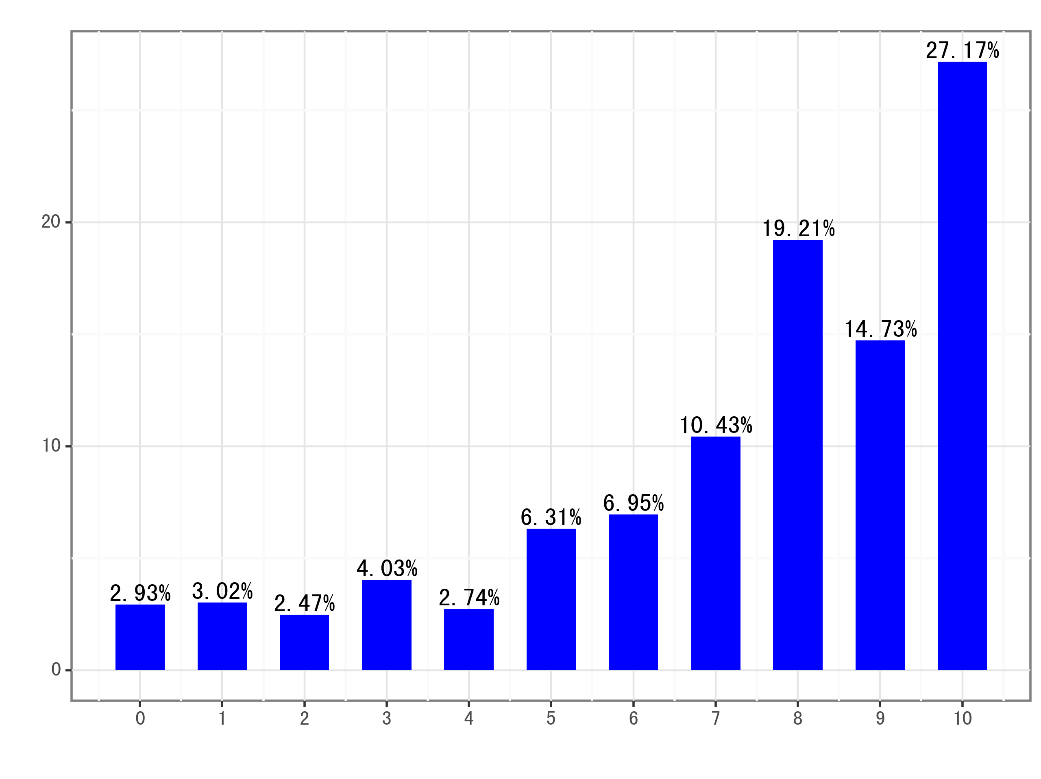


**Supplementary Figure 2.** Perception of haze hazards (the greater the value, the deeper the cognition)


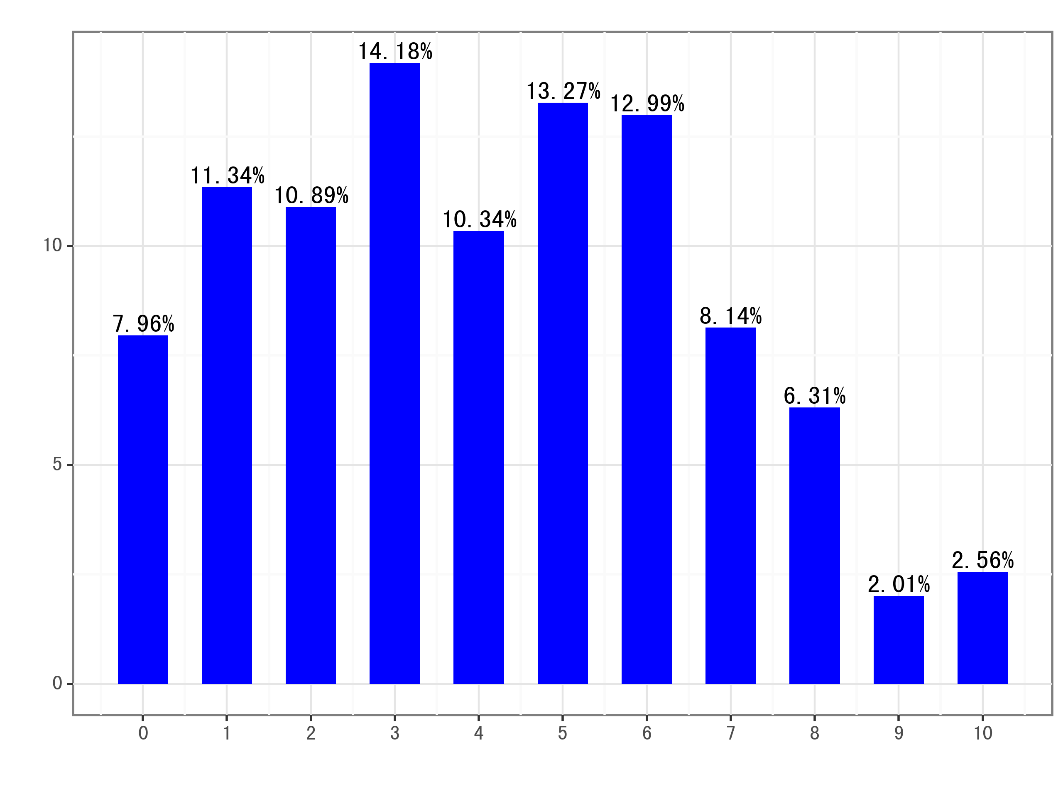


**Supplementary Figure 3.** The severity of air pollution in the area where you live (the more serious, the greater the number)


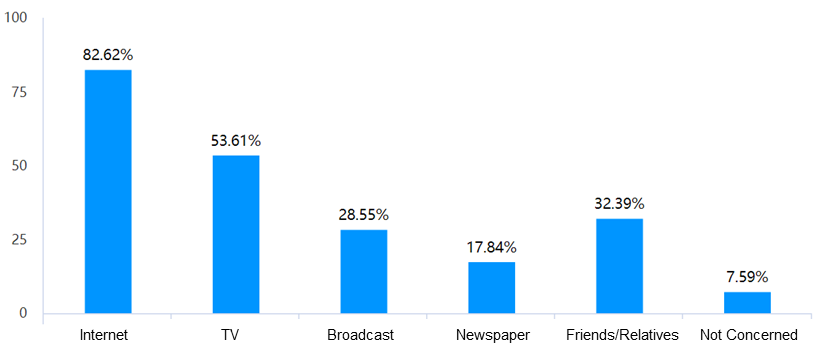


**Supplementary Figure 4.** The channel to get air pollution information


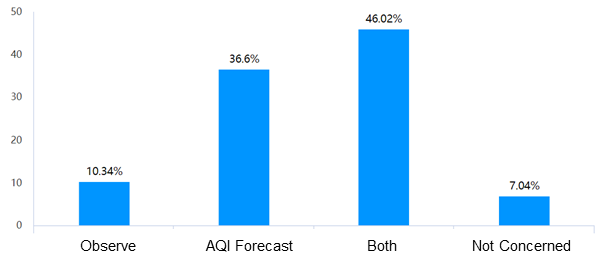


**Supplementary Figure 5.** How to get current air pollution quality information


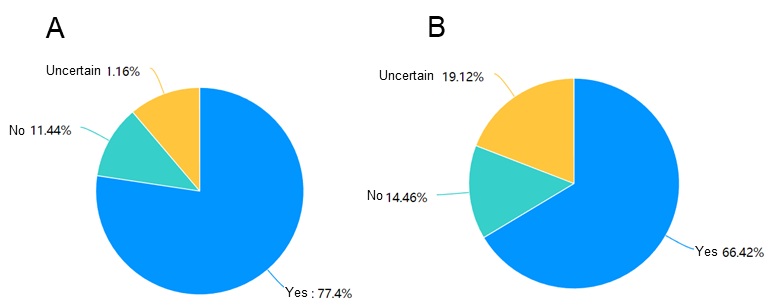


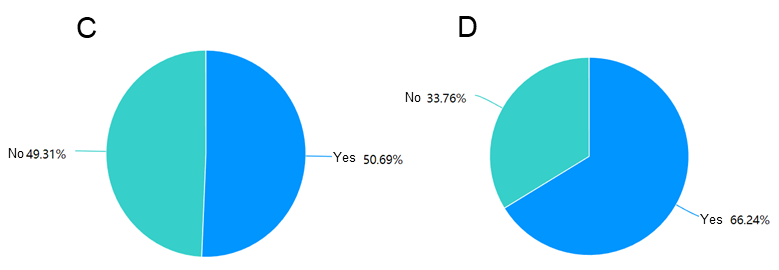


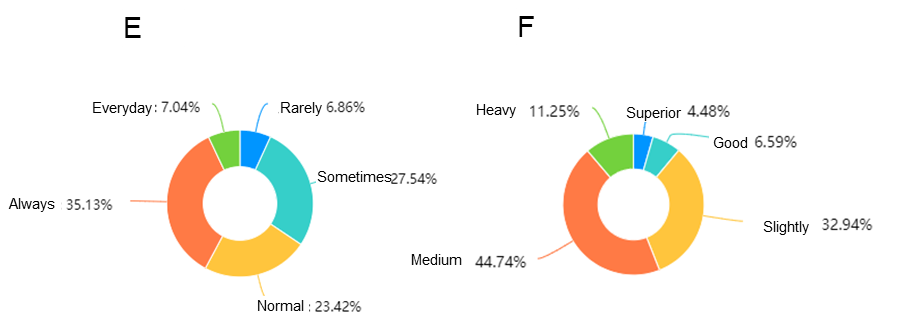


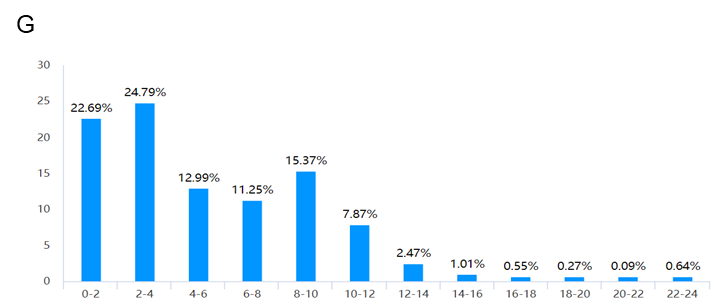
 **Supplementary Figure 6.** Statistical information of some important indicators in the questionnaire. A is whether to wear a mask outdoors when received the air pollution weather warning forecast (non-epidemic period); B is whether Canceling going out when received air pollution weather warning forecast (non-epidemic period); C is whether air purification equipment is installed in the workplace; D is whether the residential area is equipped with air purification equipment; E is whether the air pollution warning and forecast information will be paid attention to in daily life; F refers to the level of air quality above which protective measures and actions can be taken; G refers to the distribution of outdoor activity hours in different intervals; The vertical axis is the interval proportion, and the horizontal axis is the time interval.

# S6 Regional distribution of PM2.5 related premature death


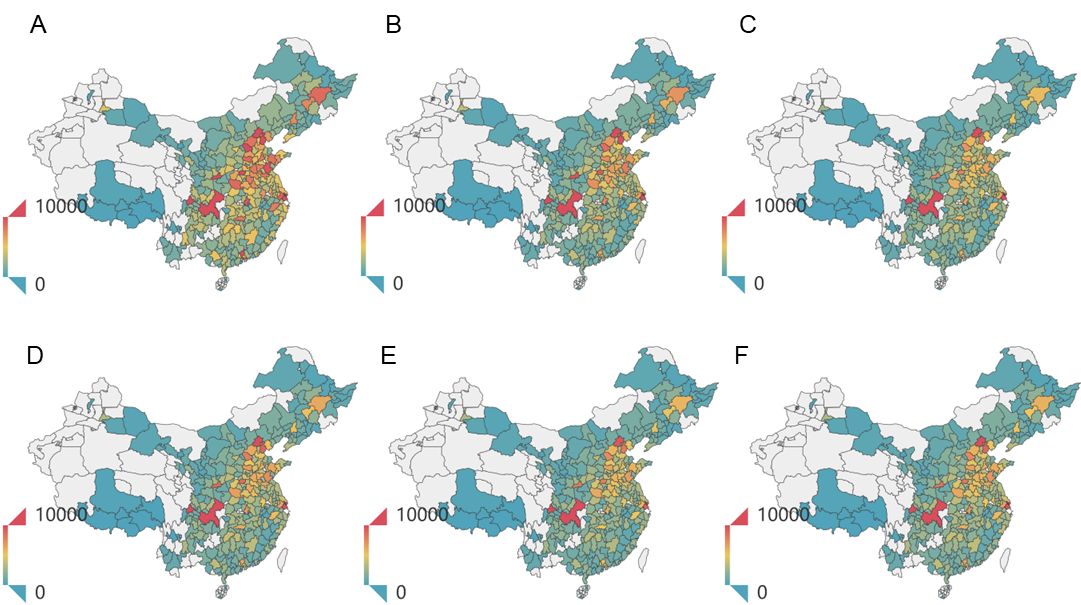


**Supplementary Figure 7**. A-F shows the spatial distribution of PM2.5-related premature deaths in 294 prefecture-level cities in 2020 under scenario S0-S5, and the lack of relevant statistical data in the blank part of the map, and the proportion of the total population in this part is less than 10%, so it is not considered. The maps were drawn by Python (v3.10, https://www.python.org/) and Pyecharts (v2.0.3, https://pyecharts.org/#/), based on the Vector Border Map of China’s City level Administrative Division in 2021 (Geographic Coordinate System: CGCS_2000).

# S7 City level protection data and inference

The cross-validation and test results of the model as Table 1 shows. The mean square error (MSE) and $R^{2}$ of the model are kept in a reasonable range. The model prediction results maintain a low error at the city level, with an average absolute error (MAE, TL) of about 0.1. The prediction results of each variable are shown in Supplementary Figure 8. In order to make the final results more stable and reliable, each variable was clipped to $\mu\pm2\delta$。$\mu$ and $\delta$ is the mean and standard deviation of each variable.

In terms of age, gender, urban and rural groups, as the number of samples of each group in each city has further decreased, it is impossible to make effective inferences. Therefore, we used the total original questionnaire to calculate the variables of each group. The specific value is shown in supplementary Table 2. As the air purification equipment can be shared by all groups in the same space, the ACR indicators of groups such as gender and age are still calculated according to the overall forecast value of each city, while the urban and rural groups are calculated according to the original questionnaire.

**Table 1.** Model training and test results

|  | Best Model | MSE(CV)^*^ | R-Square(CV)^*^ | MAE(TEST, TL)^**^ | MAE(TEST, NON-TL)^**^ |
| --- | --- | --- | --- | --- | --- |
| $ATTR_{s,i}$ | Lasso | 0.0063 | 0.7136 | 0.1009 | 0.1203 |
| $ODR_{s,i}$ | Ridge | 0.0046 | 0.7365 | 0.0408 | 0.0621 |
| $CODR_{s,i}$ | RF | 0.0053 | 0.7900 | 0.0722 | 0.0767 |
| $MR_{s,i}$ | Lasso | 0.0049 | 0.7184 | 0.1394 | 0.1699 |
| $ACR_{s,i}$ | Lasso | 0.0129 | 0.6516 | 0.1082 | 0.1121 |

*: CV is cross validate; MSE(CV) is mean square error, and the smaller the better; R-Square(CV) is fitted model’s R^2^, the bigger the better; Both of them are measured in cross validation dataset.

**: MAE is mean absolute error, which is measured in test dataset (city level data); TL is transfer learning, and NON-TL indicate that the model was not used transfer learning in training stage.


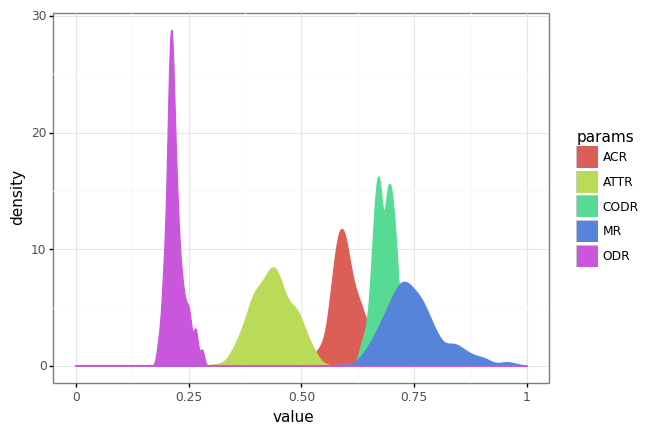


**Supplementary Figure 8.** Distribution of Predicted Values of Each Variable

**Table 2.** Variables value of each group

| Group | ODR | MR | CODR | ATTR | ACR |
| --- | --- | --- | --- | --- | --- |
| female | 0.210 | 0.788 | 0.713 | 0.428 | Yes |
| male | 0.245 | 0.756 | 0.611 | 0.413 | Yes |
| rural | 0.252 | 0.753 | 0.634 | 0.354 | 0.473 |
| city | 0.218 | 0.779 | 0.676 | 0.441 | 0.620 |
| age_0_14 | 0.227 | 0.736 | 0.566 | 0.208 | Yes |
| age_15_64 | 0.226 | 0.773 | 0.670 | 0.435 | Yes |
| age_65 | 0.223 | 0.764 | 0.706 | 0.525 | Yes |

Notes: “Yes” is set as the inferred value of each city

# S8 Search keywords

**Table 3.** Search keywords

| Natural ecological issues | ecological environment, biodiversity, land desertification, desertification, forest area, sharp reduction of forests, endangered animals, endangered plants, endangered animals and plants, animal protection, animal extinction, plant extinction, animal and plant extinction, soil erosion, indiscriminate cutting deforestation, deforestation, game, wildlife |
| --- | --- |
| Water ecological issues | water pollution, eutrophication, wastewater, pollution discharge, sewage |
| Soil ecological issues | heavy metals, waste batteries, soil pollution, organic chemical pollution, pesticides |
| Marine ecological issues | marine pollution, red tide, oil spill, marine environment, marine debris |
| Climate change issues | climate change, greenhouse effect, greenhouse gases, global warming, carbon emissions, low carbon, extreme climates, melting glaciers, sea level rise |
| Air pollution issues | air pollution, smog, acid rain, ozone layer, air pollution, air quality, pm2.5, sulfur dioxide, AQI, dust, visibility |
| Solid waste and chemical management issues | Domestic waste, solid waste, white pollution, medical waste, chemical pollution, industrial waste, garbage sorting |
| Other | environmental protection, garbage, environmental, protection, pollutants |

# S9 Comparison of results between IER and GEMM

In order to investigate the robustness of our study under different premature death computational models, we used the GEMM model as an example to recalculate some of the results in this study. The GEMM model is an updated version of the IER model proposed by Burnett et al.^15^ in 2018. Compared with the IER model, GEMM has added a new queue study on PM2.5 related premature death and relaxed the limitations of the IER model. This makes the GEMM model more applicable. The risk function of the GEMM model is shown in formulas (5)-(9).

$$\begin{aligned} R\left( z \right)=e^{\theta T\left( z \right)}\#\left( 5 \right) \end{aligned}$$

$$\begin{aligned} T\left（ z \right）=f\left( z \right)w\left( z \right)\#\left( 6 \right) \end{aligned}$$

$$\begin{aligned} f\left( z \right)=log\left( 1+\frac{z}{\alpha} \right)\#\left( 7 \right) \end{aligned}$$

$$\begin{aligned} w\left( z \right)=\frac{1}{1+e^{\frac{-\left( z-\mu\right)}{v}}}\#\left( 8 \right) \end{aligned}$$

$$\begin{aligned} z=\left\{ \begin{aligned} p-2.4, p>2.4 \\ 0, p<=2.4 \end{aligned} \right.\#\left( 9 \right) \end{aligned}$$

With p is the exposure concentration of PM2.5 (µg/m^3^). $\alpha$, $\mu$, v, $\theta$ are the fitting parameters of the model in cohort studies. The parameter values are set in the Supplementary Material section of Burnett et al.'s research. Keeping the program for calculating the number of premature deaths in this study unchanged, simply replacing the IER risk function with the GEMM risk function during the process can obtain the new distribution of premature deaths in different scenarios. For the sake of comparison, the GEMM model in this study still only calculates the number of deaths associated with four diseases.

Supplementary Figures 9-10 and Figures 5A-B (Figure 6A-B) in the original text indicate that there are two main differences between the GEMM model and the IER model used in this study: firstly, there is a significant increase in the number of premature deaths in each scenario; Secondly, there has been a certain change in the composition of disease related deaths in each scenario (IHD has significantly increased, surpassing Stroke as the primary associated disease). However, the overall distribution pattern of premature deaths in different scenarios is completely the same for the two models. This is because the core content of this study is to calculate the impact of risk information on the equivalent PM2.5 concentration through the I-BEPEM model, while the core content of models such as IER and GEMM mainly focuses on the relative risk values of various diseases at different concentrations. That is to say, the core content of our research and the risk calculation model for premature death are decoupled. This means that using other models such as GEMM and MR-BRT will not invalidate any conclusions in this study. The only thing that changes is the absolute quantity. Table 2 summarizes the absolute values of the total number of premature deaths under different scenarios for these two models.


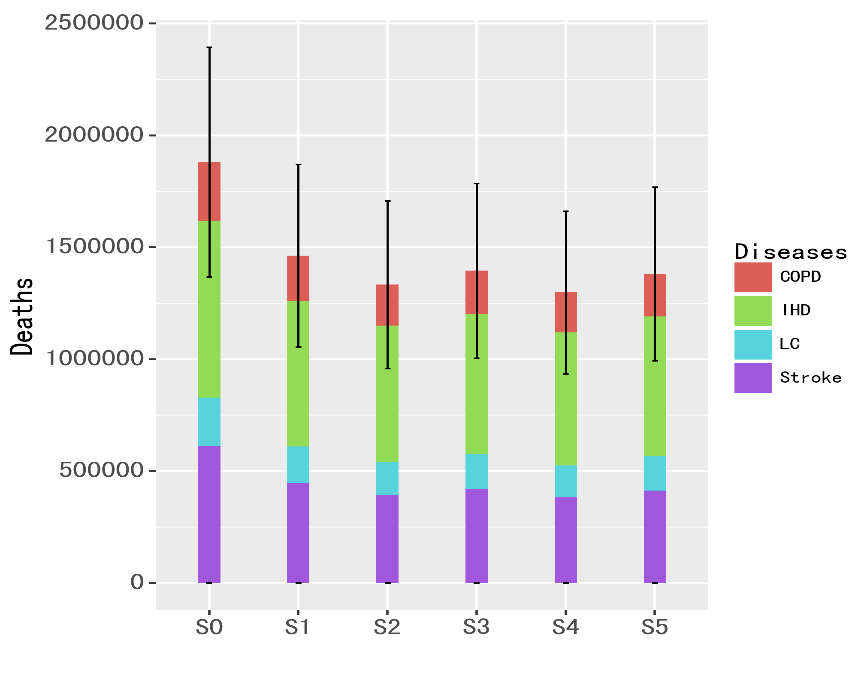


Supplementary Figure 9


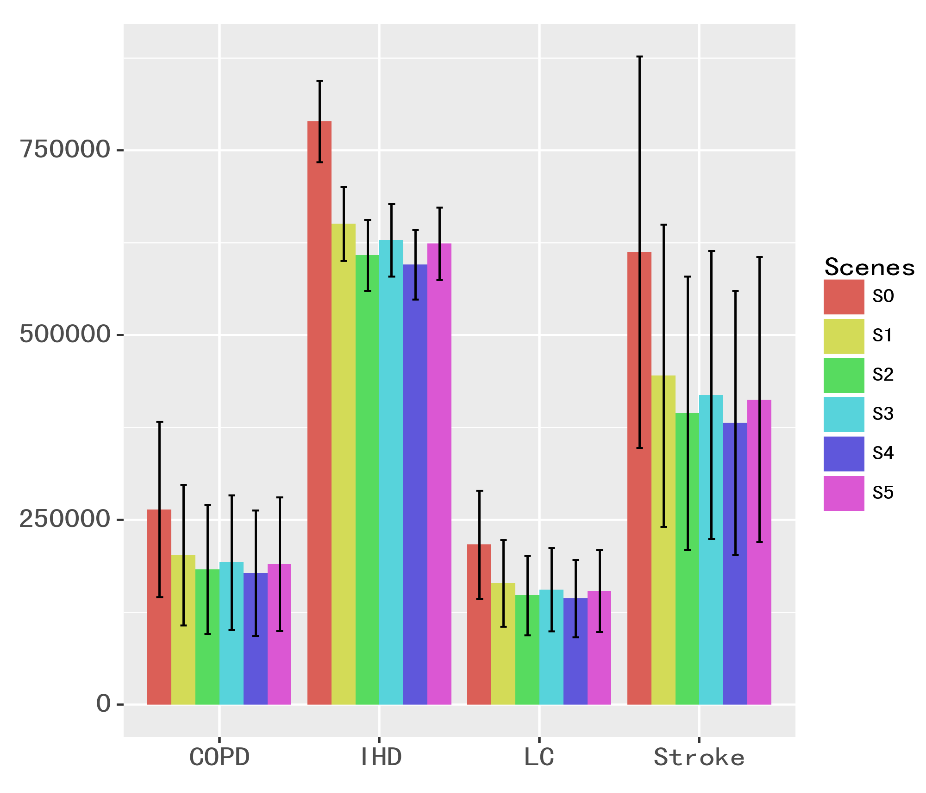


**Supplementary Figure 10**

Table 4

| scenarios | IER deaths (million) | GEMM deaths (million) |
| --- | --- | --- |
| S0 | 95.8 | 188.3 |
| S1 | 71.5 | 146.2 |
| S2 | 63.3 | 133.2 |
| S3 | 67.4 | 139.5 |
| S4 | 60.7 | 129.8 |
| S5 | 66.5 | 138.1 |

# S10 AQI standard of China and USA

Table 5

| AQI | Average PM2.5 concentration (μg/m^3^) | | Air quality level | |
| --- | --- | --- | --- | --- |
|  | China | USA | China | USA |
| 0-50 | 0-35 | 0-12 | excellent | good |
| 50-100 | 35-75 | 12-35 | good | moderate |
| 100-150 | 75-115 | 35-55 | lightly polluted | unhealthy for sensitive groups |
| 150-200 | 115-150 | 55-150 | moderately polluted | unhealthy |
| 200-300 | 150-250 | 150-250 | heavily polluted | very unhealthy |
| 300-500 | 250-500 | 250-300 | severely polluted | hazardous |

# References

1. Fischhoff, B., Lichtenstein, S., Slovic, P., Derby, S. & Keeney, R. *Acceptable Risk*, (Cambridge University Press, Cambridge UK, 1981).

2. Association, C.T.C. Group standard of PM2.5 Protective Mask (TAJ 1001-2015). *Textile testing and standards* **2**, 50 (2016).

3. Xiang, J.*, et al.* Reducing Indoor Levels of "Outdoor PM2.5" in Urban China: Impact on Mortalities. *Environmental Science & Technology* **53**(2019).

4. Wendi, L., Chun, Z., construction, R., Jinyu, X. & Fang, L. Analysis of indoor air purification effect of air purifier. *Green Building* **7**, 58-60 (2015).

5. Dayu, H.*, et al.* Evaluation on the purification effect of household air purifiers in winter heating period in Beijing. *Environmental and Occupational Medicine* **35**, 33-38 (2018).

6. Xiufeng, Y., Limei, X. & Xuehan, Z. Measurement and Analysis of Air Purifier Performance. *Journal of Xi'an University of Architecture and Technology (Natural Science Edition)* **51**, 757-762 (2019).

7. Liu, L.*, et al.* Removal of indoor PM2.5 by air purifier - taking Dalian as an example. in *The 7th National Conference on Environmental Chemistry* 49-50 (Guiyang, Guizhou, China, 2013).

8. Burnett, R.*, et al.* An Integrated Risk Function for Estimating the Global Burden of Disease Attributable to Ambient Fine Particulate Matter Exposure. *Environmental health perspectives* **122**(2014).

9. Pan, S.J. & Qiang, Y. A Survey on Transfer Learning. *IEEE Transactions on Knowledge and Data Engineering* **22**, 1345-1359 (2010).

10. Li, Y. On incremental and robust subspace learning. *Pattern Recognition* **37**, 1509-1518 (2004).

11. Sun, B., Feng, J. & Saenko, K. Return of Frustratingly Easy Domain Adaptation. *AAAI Press* (2015).

12. Shen, H.*, et al.* Increased air pollution exposure among the Chinese population during the national quarantine in 2020. *Nature Human Behaviour*.

13. Yanan, L.H.T.L.L. Influence of indoor ventilation mode on PM2.5 concentration. *Journal of Shandong Agricultural University (Natural Science Edition)* **52**, 338-342 (2021).

14. Laboratory, N.E.R. Estimating contributions of outdoor fine particles to indoor concentrations and personal exposures: Effects of household characteristics and personal activities. (ed. EPA, U.S.) (National Exposures Research Laboratory, USA, 2006).

15. Burnett, R.*, et al.* Global estimates of mortality associated with long-term exposure to outdoor fine particulate matter. *Proc Natl Acad Sci U S A* **115**, 9592-9597 (2018).
